# Ubiquitin Fold Modifier 1 (UFM1) and Its Target UFBP1 Protect Pancreatic Beta Cells from ER Stress-Induced Apoptosis

**DOI:** 10.1371/journal.pone.0018517

**Published:** 2011-04-06

**Authors:** Katleen Lemaire, Rodrigo F. Moura, Mikaela Granvik, Mariana Igoillo-Esteve, Hans E. Hohmeier, Nico Hendrickx, Christopher B. Newgard, Etienne Waelkens, Miriam Cnop, Frans Schuit

**Affiliations:** 1 Gene Expression Unit, Department Molecular Cell Biology, KatholiekeUniversiteit Leuven, Leuven, Belgium; 2 Laboratory of Experimental Medicine, Université Libre de Bruxelles, Brussels, Belgium; 3 Sarah W. Stedman Nutrition and Metabolism Center and Departments of Pharmacology and Cancer Biology and Medicine, Duke University Medical Center, Durham, North Carolina, United States of America; 4 Prometa, Department Molecular Cell Biology, Katholieke Universiteit Leuven, Leuven, Belgium; 5 Division of Endocrinology, Erasmus Hospital, Brussels, Belgium; Institut Pasteur, France

## Abstract

UFM1 is a member of the ubiquitin like protein family. While the enzymatic cascade of UFM1 conjugation has been elucidated in recent years, the biological function remains largely unknown. In this report we demonstrate that the recently identified C20orf116 [1], which we name UFM1-binding protein 1 containing a PCI domain (UFBP1), andCDK5RAP3 interact with UFM1. Components of the UFM1 conjugation pathway (UFM1, UFBP1, UFL1 and CDK5RAP3) are highly expressed in pancreatic islets of Langerhans and some other secretory tissues. Co-localization of UFM1 with UFBP1 in the endoplasmic reticulum (ER)depends on UFBP1. We demonstrate that ER stress, which is common in secretory cells, induces expression of *Ufm1*, *Ufbp1* and *Ufl1* in the beta-cell line INS-1E.siRNA-mediated *Ufm1* or *Ufbp1*knockdown enhances apoptosis upon ER stress.Silencing the E3 enzyme UFL1, results in similar outcomes, suggesting that UFM1-UFBP1 conjugation is required to prevent ER stress-induced apoptosis. Together, our data suggest that UFM1-UFBP1participate in preventing ER stress-induced apoptosis in protein secretory cells.

## Introduction

Ubiquitin is a small (8.5 kDa) protein, which is evolutionary conserved in eukaryotes. The so-called post-translational modification ‘ubiquitilation’ is the covalent binding of ubiquitin to a substrate protein. The best-known function of ubiquitilation is the targeting of proteins for degradation by the proteasome. However, ubiquitilation can also affect subcellular localization, interactions, stability or activity of the substrate protein [2]. Therefore, ubiquitin can participate in a wide variety of cellular processes. Besides ubiquitin, a large family of ubiquitin-like proteins (Ubls) has been identified. These proteins do not necessarily share a high degree of sequence similarity to ubiquitin, but they all contain the typical ubiquitin-like tertiary structure [3].

Ubiquitin-fold modifier 1 or UFM1 has recently been identified as a novel protein-conjugating system, displaying a similar tertiary structure to ubiquitin [4]. To be activated, UFM1 is processed C-terminally by two specific proteases, UfSP1 and UfSP2 [5], [6]. After processing, UFM1 is activated via the E1 enzyme, UBA5, and then conjugated by the E2 enzyme, UFC1. UFL1 has very recently been identified as the E3 enzyme and C20orf116 as a substrate of UFM1 [1]. However, cellular functions associated withtarget proteins that aremodified by UFM1 are still unknown.

The pancreatic beta cell is unique in its capacity to synthesize, store and secrete insulin with precise rates to cover the metabolic needs of the organism [7]. The fine-tuning of insulin synthesis, storage and secretion is regulated at many levels of gene expression, ranging from transcription, mRNA stability to translation and folding [8]. Microautophagic activity also plays a role in maintaining cellular hormone stores to optimal levels [9]. To fulfill this heavy task of insulin biosynthesis, the beta cell has a highly developed endoplasmic reticulum (ER). Optimal functioning of the ER is essential for proper protein folding and cell survival. Any disturbance in ER folding needs and capacity leads to ER stress and activation of the ER stress response (also called unfolded protein response) [10], [11], [12], [13]. The aim of this response is to restore ER homeostasis and at least three functionally distinct responses have been identified. First, up-regulation of ER chaperones to increase protein folding activity and to prevent protein aggregation [14]. Second, attenuation of global protein translation to reduce the load of newly synthesized proteins and prevent excessive accumulation of unfolded proteins [15], [16]. Finally, degradation of proteins misfolded in the ER, which is called ER-associated degradation (ERAD) [17]. Three ER stress transducers can be recognized: IRE1, ATF6 and PERK [15], [18], [19]. IRE1 induces *Xbp1* splicing, which in turn, together with ATF6, induces transcription of chaperones (e.g. BiP), genes involved in ERAD and CHOP. In parallel, PERK activation upon ER stress increases eIF2α phosphorylation, which on the one hand inhibits protein translation and on the other hand activates transcription of chaperones, genes involved in ERAD and CHOP. When this ER stress response fails to restore ER homeostasis, apoptosis is triggered [20].

The link between ER dysfunction and diabetes has been studied extensively. PERK null mice have increased beta cell apoptosis and early onset diabetes [21], eIF2α^S51A^ heterozygous mice develop diabetes when fed a high fat diet [22], and CHOP^−/−^mice have improvedbeta cell function and bettercell survival in conditions that cause diabetes in control mice [23]. The conservation of these regulatory pathways among vertebrates and the link between PERK mutations and diabetes in patients with the Wolcott-Rallison syndrome [24] indicate that ER stress is important for diabetes in humans (reviewed in [11]).

In the present study we have investigated the UFM1 pathway in rodent pancreatic beta cells using both mouse isolated islets and the cell lines INS1 and MIN6. Our results show thatUFM1 and its target UFBP1are highly expressed in the pancreatic islets of Langerhans, and that their expression is increased upon ER stress.We provide evidence that UFM1 and UFBP1are important for the prevention of ER stress-induced apoptosis.

## Results

### Ufm1 is highly expressed in pancreatic islets of Langerhans

**Figure 1:**
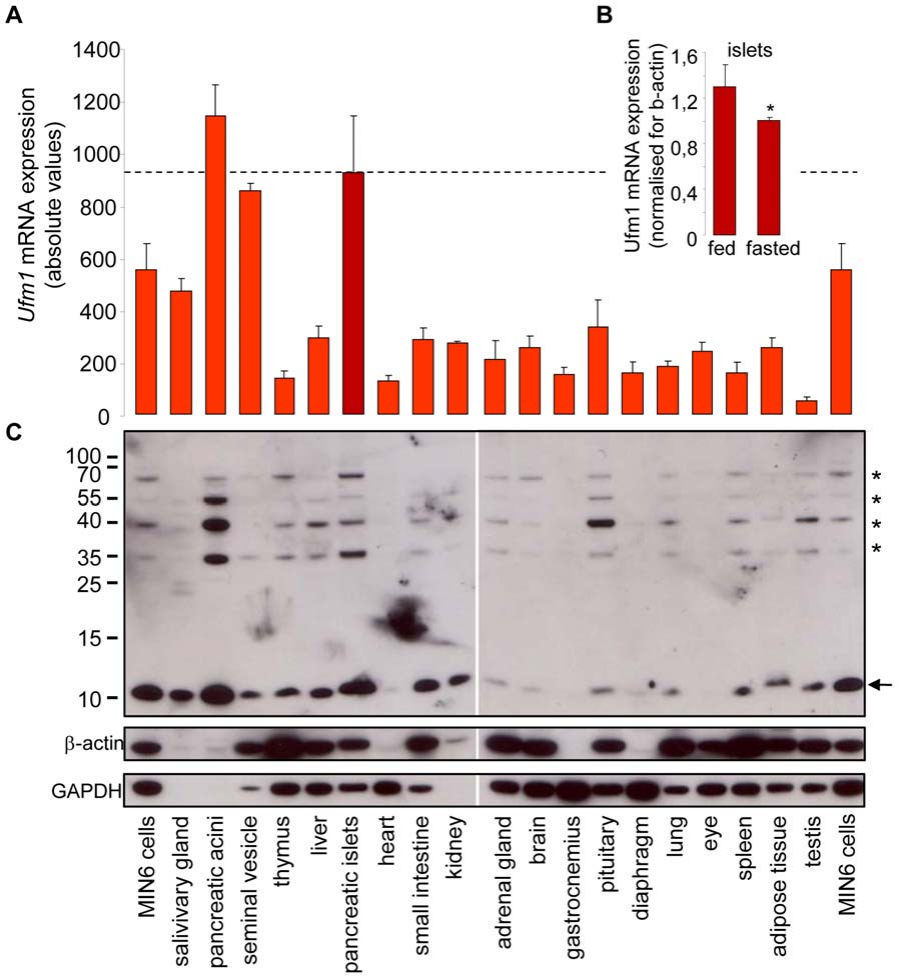
Expression profile of *Ufm1* in differentmouse tissues. A *Ufm1* mRNA expression in 19 different mouse tissues and MIN6 cells, measured via microarray (probe set 1449263_at), n≥3, B *Ufm1* mRNA expression in islets from mice which were fasted for 20 hours or fed a normal diet, n≥3, * p = 0.02, C UFM1 protein expression in the same mouse tissues. Immunodetection was done with a UFM1 specific antibody. Both free UFM1 (←) and UFM1 conjugates (*) are shown. An equal amount of protein was loaded on gel. Both GAPDH and β-actin were used as control, since no protein is equally expressed in all tissues. Representative immunoblot is shown.

Both in microarray mRNA expression analysis in the mouse (Figure 1A)and via quantitative real-time PCR, using *Ufm1*-specific primers and probe (Figure S1)the transcript encoding *Ufm1* (Ubiquitin-fold modifier 1) was found to be very abundant in protein-secreting cells, especially pancreatic acini, islets of Langerhans and salivary glands. Furthermore, *Ufm1* mRNA levels in islets were higher in fed mice, as compared to mice that were fasted for 20 hours (Figure 1B). A similar tissue distribution was observed at the protein level, using a UFM1-specific antibody (Figure 1C). Not only free UFM1 could be detected, but also several UFM1 conjugates. From this tissue expression profile we hypothesized that UFM1 plays an important role in protein secreting cells like beta cells in the islets of Langerhans.

### Identification of the interactions between UFM1, UFBP1, CDK5RAP3 and UFL1

To identify the target(s) of UFM1, we performed a UFM1 affinity purification. We engineered a STrEP-tag at the N-terminus of UFM1and transfected clonal insulin-producing MIN6 cells with this STrEP-*Ufm1* construct. We exposed the cells for 2 hours to 10 mg/l cycloheximide to increase UFM1 conjugation (see below). STrEP-UFM1 was affinity purified and the eluates were analyzed via SDS-PAGE and coomassie staining (Figure S2A). In total, 9 protein fragments were eluted from gel and further analyzed via mass spectrometry (Table 1). We identified the conjugating enzyme UFC1 in the∼20 and ∼36 kDa fragment and the activating enzyme UBA5 in the ∼45 kDa and ∼60 kDa protein fragments [4]. Also the very recently reported ligating enzyme UFL1 (∼100 kDa fragment; 1810074P20Rik) and the substrate C20orf116 (∼40 kDa fragment; 2600009E05Rik) [1] were picked-up in this screen. CDK5RAP3/LZAP (∼60 kDa fragment), two heat shock proteins HSPA8 and HSPA5 (BiP) (70 kDa fragment) and pyruvate carboxylase (∼130 kDa) were the other identified proteins. The isolation of pyruvate carboxylase is perhaps not surprisingly since it is biotinylated and highly expressed in beta cells [25].

**Table 1:** List of the identified proteins by mass spectrometry after Ufm1_STrEPtag affinity purification.

Frag-ment	protein name	Swiss ProtAccession nr	Mw (kDa)	# peptides seq/unique		sequence coverage %	Function
1	UFM1; Ubiquitin fold modifier 1	P61961	9	2	2	31.8	ER stress-induced apoptosis (this paper)
2	UFC1; Ufm1-conjugating enzyme 1	Q9CR09	20	3	2	10.8	E2 enzyme of UFM1 (Komatsu et al, 2004)
3	UFC1; Ufm1-conjugating enzyme 1	Q9CR09	20	2	1	6	E2 enzyme of UFM1 (Komatsu et al, 2004)
4	UFBP1; Ufm1 binding protein containing a PCI domain	Q80WW9	36	4	3	13.3	ERAD/ER stress-induced apoptosis, target of UFM1 (Tatsumi et al, 2010; this paper)
5	UBA5; Ubiquitin-like modifier-activating enzyme 5	Q8VE47	45	2	2	4.7	E1 enzyme of Ufm1 (Komatsu et al, 2004) [4]
6	UBA5; Ubiquitin-like modifier-activating enzyme 5	Q8VE47	45	2	2	5.2	E1 enzyme of UFM1 (Komatsu et al, 2004)
	CDK5RAP3; CDK5 regulatory subunit-associated protein 3	Q99LM2	57	4	3	6.4	tumor suppressor (Wang et al, 2007); substrate of Ufm1 (this paper)
7	HSPA5/BiP; 78 kDa glucose-regulated protein precursor	P20029	72	3	3	4.7	ER stress, chaperone (Ma, Hendershot, 2004)
	HSPA8; Heat shock 70 kDa protein 8	P63017	71	5	5	8	chaperone (Zimmerman, 1998)
8	Ufl1; UFM1 ligation protein	Q8CCJ3	100	7	7	9.7	E3 enzyme of UFM1 (Tatsumi et al., 2010, this paper)
9	PCX;Pyruvate carboxylase	Q05920	130	12	11	12.3	anaplerosis//cataplerosis (Fransson et al, 2006)

**Figure 2:**
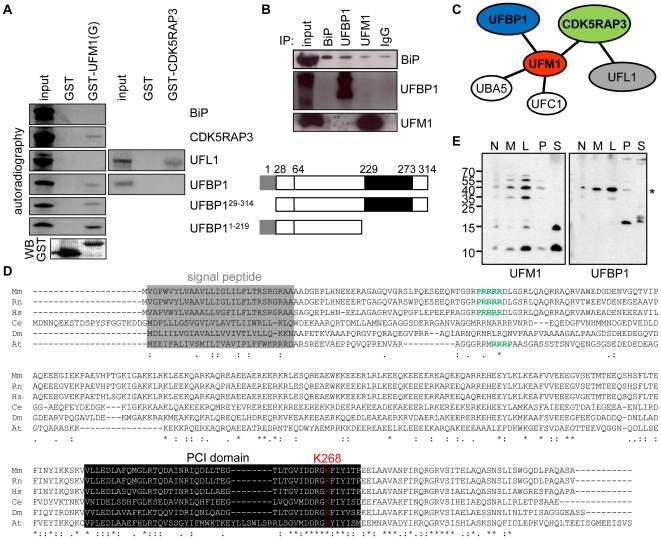
UFM1 interacts with UFBP1 and CDK5RAP3. A GST pull down (UFM1(G) and CDK5RAP3) with *in vitro* T7 transcribed/translated ^35^S-labelled UFBP1, UFBP1^29–314^, UFBP1^1–219^ CDK5RAP3, UFL1 and BiP. Lane 1 shows the starting labeled proteins used for the pull down experiment (input) and a schematic overview of the used UFBP1 constructs. A GST-antibody was used for immunoblotting (WB), B co-immunoprecipitation with BiP, UFL1 and UFM1 specific antibodies, C Schematic overview of the protein interactions of UFM1 demonstrated in mouse in this manuscript, D Evolutionary conservation of UFBP1 (2600009E05Rik). Protein sequence in Mm (Musmusculus), Rn (Rattusnovergicus), Hs (Homo sapiens), Dr (Daniorerio), Gg (Gallus gallus), Tn (Tetraodonnigroviridis), Fr (Fuguribripes), Ag (Anopheles gambiae), Ce (Caenorhabditiselegans), Dm (Drosophila melanogaster) and At (Arabidopsis thaliana). Alignment was performed using ClustalW. The signal peptide of UFBP1 is boxed in grey, the nuclear localization signal is depicted in green, the PCI domain is boxed in black and lysine268 (K268) is shown in red, E Presence of UFM1 conjugates at the same height and with the same cellular localization as UFBP1 (*). MIN6 cell lysates were separated in 5 different fractions: N = nuclear and whole cell fraction (770×*g*), M = heavy mitochondrial fraction (2330×*g*), L = light mitochondrial, peroxisomal and lysosomal fraction (13,000×*g*), P = cell membrane fraction (100,000×*g*) and S = cytosolic and large protein complexes supernatant (100,000×*g*).

The interactions between the identified proteins and UFM1 were further analyzed by GST pull down. A GST-tag was coupled to the N-terminus of mouse UFM1 with a C-terminal ending glycine residue (GST-UFM1(G)). Purified GST-UFM1(G) and GST protein were coupled to glutathione-agarose beads and incubated with ^35^S-labeled mouse 2600009E05Rik/C20orf116, CDK5RAP3, BiP and 1810074P20Rik /UFL1, which were generated by T7 *invitro* transcription/translation. 2600009E05Rik and CDK5RAP3 were recovered from the GST-Ufm1(G) coupled beads, but not from the GST coupled beads (Figure 2A). Therefore, we propose to name 2600009E05Rik/C20orf116asUFBP1, or UFM1 binding protein 1 containing a PCI domain. Although very weak, the interaction between UFM1 and UFBP1 was also confirmed via co-immunoprecipitation with a UFBP1 and UFM1 specific antibody (Figure 2B). Neither BiP nor UFL1 could bind to GST-UFM1(G) or GST coupled beads, indicating that they do not interact directly with UFM1. Co-immunoprecipitation with a BiP or UFM1 specific antibody could also not demonstrate a binding between UFM1 and BiP (Figure 2B). However, an interaction between UFBP1 and BiP was observed after co-immunoprecipitation with a UFBP1 specific antibody (Figure 2B). To analyze why UFL1 did not bind in our *in vitro* screen, we used the same GST pull down strategy, using CDK5RAP3-GST as bait. Figure 2A shows an interaction between CDK5RAP3 and UFL1, but not with UFBP1. These results show that UFM1 directly binds (covalent or non-covalent) to UFBP1 and CDK5RAP3, and that UFL1 binds to CDK5RAP3 (Figure 2C). Recently, the interaction between UFL1 and CDK5RAP3 was also demonstrated by some other groups [26], [27], [28].

**Figure 3:**
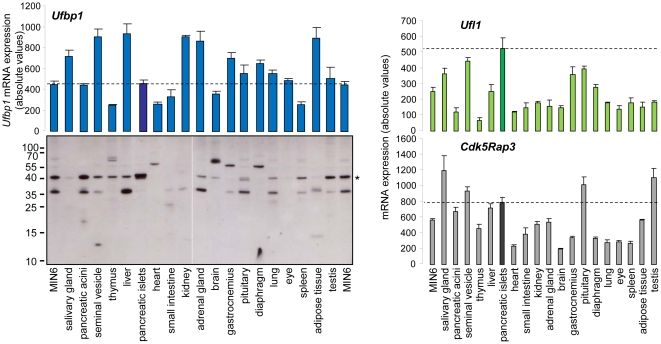
mRNA expression of *Ufbp1*, *Ufl1* and *Cdk5Rap3* in 23 different mouse tissues and MIN6 cells, measured via microarray (probe set 1434702_at, 1429008_at and 1423067_at, respectively). UFBP1 protein expression measured in the same tissue panel. A UFBP1 specific antibody was used, and the same amount of protein was loaded as in Figure 1C.

We also analyzed the mRNA expression of the different interacting partners of UFM1 in a mouse tissue panel via microarray (Figure 3). While *Ufbp1* mRNA expression levels were rather homogenous in the different mouse tissues, the protein UFBP1 showed the highest expression in pancreatic islets, followed by pancreatic acini and testis. *Ufl1* showed the highest expression in the pancreatic islets, followed by some other secretory tissues e.g. seminal vesicle and salivary gland and *Cdk5Rap3* was highest expressed in salivary gland and testis, followed by seminal vesicle, pituitaryand pancreatic islets.

UFBP1 is, like UFM1, evolutionary conserved. The protein is present in plants, invertebrates and vertebrates (Figure 2D). Detailed computational analysis of the UFBP1 protein (314 aa) sequence and structure revealed the presence of a signal peptide for the ER (aa 1–28), a transmembrane helix (aa 5–22), a nuclear localization signal (aa 65–69) and a PCI domain (aa 229–273) [29]. PCI domains are present in several regulatory Proteasome subunits, COP9 subunits, eIF3 translation initiation factor subunits, and in certain other multi-protein complexes [30]. To investigate the role of these two domains in the binding with UFM1, we performed a new GST-pull down experiment. We first constructed a*Ufbp1* construct without the signal peptide, UFBP1^29–314^, or without the PCI domain, UFBP1^1–219^. ^35^S-labelled UFBP1^29–314^ (without the signal peptide) and UFBP1^1–219^ (without the PCI domain) were generated by T7 *invitro* transcription/translation. Figure 2A shows that both truncated proteins could still bind to UFM1 *in vitro*, showing that neither the signal peptide nor the PCI domain is required for the interaction between UFM1 andUFBP1.

To clarify if the UFM1/UFBP1-interaction is covalent or non-covalent, we first performed a STrEP-tag affinity purification with UFBP1_STrEP, similar to the affinity purification with STrEP_UFM1. The eluates were also analysed via SDS-PAGE and Coomassie staining (Figure S2B). Three distinct protein fragments (∼38, ∼41 and ∼50 kDa) were identified as UFBP1. Interestingly, the protein fragment of ∼50 kDa was about 10 kDa too high for UFBP1 and missed the peptides containing the unmodified K268. The ∼10 kDa shift is in perfect agreement with a UFM1 modification (+9.1 kDa) of UFBP1 and lysine K268 being involved in UFM1 conjugation with UFBP1 is in agreement with previous published data [1]. In a second approach, we performed cellular fractionation of MIN6 lysates and Figure 2E clearly shows that the UFM1 conjugate of about 40 kDa was at the same height and showed the same cellular localization as UFBP1. In addition, overexpression of UFBP1_eGFP, resulted in a UFM1 conjugate of ∼70 kDa (Figure S2C). Surprisingly, also overexpression of UFBP1^K268R^-eGFP resulted in a UFM1 conjugate of ∼70 kDa (Figure S2C). These data indicate that UFM1 can bind covalent to UFBP1 and that K268 is involved, but not required for this binding.

### Translocation of UFM1 to the ER depends on UFBP1

**Figure 4:**
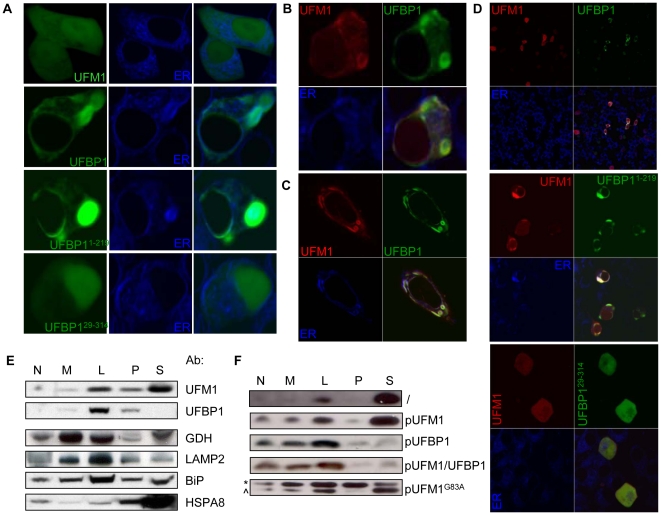
UFBP1 and UFM1 are co-localized in the ER. A INS1-832/13 cells transfected with different eGFP constructs as indicated on the picture. INS1-832/13 cells (B) and Hela cell (C) co-transfected with mRFP-UFM1 and UFBP1-eGFP, D Overview of INS1-832/13 cells co-transfected with mRFP-UFM1 and UFBP1-eGFP, UFBP1^29–314^-eGFP or UFBP1^1–214^-eGFP, as depicted. Cells were also stained with an ER-tracker (blue). Pictures were taken with a 63× objective on a Zeiss confocal microscope, E UFM1 and UFBP1 expression in different MIN6 cellular fractions, similar as in Figure 2E. Different cellular markers were used: GDH, mitochondrial marker; LAMP2, lysosomal marker; BiP, ER marker and HSPA8, cytosolic marker. **F** UFM1 expression after cellular fractionation of MIN6 cells overexpressing UFM1, UFBP1, both UFM1 and UFBP1 or UFM1^G83A^, as depicted (*, unprocessed; ∧, processed).

Different eGFP and mRFP fusion constructs were made and transfected into INS1-832/13 cells, to analyze the cellular localization of UFM1 and UFBP1 via fluorescence microscopy. After overexpression, UFM1 was equally localized in the cytoplasm and the nucleus (Figure 4A). Overexpression of full length UFBP1-eGFP showed an ER-specific expression. Deletion of the PCI domain of UFBP1 had almost no effect on the localization of the protein, while deletion of the signal peptide resulted in an exclusive nuclear localization (Figure 4A). When we overexpressed mRFP-UFM1 and UFBP1-eGFP together, UFBP1 remained localized in the ER, but UFM1 was no longer equally distributed over cytoplasm and nucleus (Figure 4B,D). Instead, it was more localized in the ER, indicating that overexpression of UFBP1 influences the localization of UFM1. A similar localization pattern was observed in cells where mRFP-UFM1 and UFBP1^1–219^-eGFPwere co-expressed (Figure 4D, Figure S3B). Overexpression of UFM1 together with UFBP1^29–314^, which is truncated for the signal peptide, however, resulted primarily in a nuclear localization of UFM1 (Figure 4D, Figure S3B). The same results were obtained after overexpression of these constructs in human HeLa cells (Figure 4C, Figure S3A), indicating that UFM1 and UFBP1 localization is similar in mouse and human. Next we overexpressed the mutants UFBP1^K268R^ and UFM1^G83A^ in INS1 cells. Processing of UFM1 to its mature form is significantly reduced in the UFM1^G83A^ mutant [1]. Both mutants showed a similar localization as their WT counterpart (Figure S4A and results not shown). Overexpression of mRFP-UFM1 and UFBP1-eGFP in INS1 cells resulted in 34±4% of the cells in co-localization of UFM1 and UFBP1 in the ER. When the mutant UFBP1^K268R^-eGFP was overexpressed together with WT UFM1, we found co-localization in 33±2% of the cells, which is similar with WT UFBP1. After co-transfection of UFM1^G83A^-mRFP and UFBP1-eGFP or UFBP1^K268R^-eGFP, the amount of cells with co-localization of the two proteins was significantly increased (70±7%; p = 0.006 and 60±6%; p = 0.02, respectively, n = 4) (Figure S4B), compared to overexpression of the WT proteins.

To ensure that overexpression did not cause a mislocalization of the proteins, we also detected the cellular localization of endogenous UFM1 and UFBP1 via cellular fractionation. It is clear that UFBP1 was present in the same fraction as BiP, indicative for ER localization (Figure 4E). UFM1 partially co-localized with UFBP1 in the ER, but a significant amount of UFM1 protein was detected in the cytosolic fraction, which contains both cytosolic proteins and large protein complexes. Overexpression of UFM1 did not change the localization of UFM1, still cytoplasmic and ER (Figure 4F). In contrast, when UFBP1 or both, UFM1 and UFBP1 were overexpressed, UFM1 was mainly expressed in the ER (Figure 4F), similar to what we observed via immunocytochemistry. Overexpression of UFM1^G83A^ resulted in a wrong localization when not processed, but a normal localization was observed when processed (Figure 4F, upper and lower band respectively). However, via fluorescence microscopy, UFM1 and UFM1^G83A^ showed the same localization and UFM1^G83A^ was still partially co-localized in the ER when overexpressed with UFBP1 (Figure S4).

These data indicate that UFBP1 and UFM1 are partially co-localized in the ER and that UFBP1 plays an important role in the compartmentalization of UFM1 in the cell. We also show that the PCI domainof UFBP1, containing lysine K268, and glycine G83 of UFM1 are not required for this co-localization, suggesting that a non-covalent binding between UFM1 and UFBP1 could be responsible for the co-localization in the ER.

### UFM1 and UFBP1 are not involved in glucose stimulated insulin secretion

Based on the expression profile of UFM1and its cellular localization, we hypothesized that UFM1 could play a role in the secretory pathway of protein secreting cells. To address this, we investigated the role of UFM1 and UFBP1 on insulin secretion in the rat glucose-responsive insulinoma cell line INS1-832/13. The effect of Ufm1 specific RNAi mediated silencing on glucose-stimulated insulin release was examined 48 hours after transfection of 832/13 cells with a*Ufm1*- or *Ufbp1*-specific siRNA duplex or a control duplex (siControl) with no known sequence homology. Treatment with the *Ufm1* or *Ufbp1*siRNA duplex caused respectively a ∼60% or 80% decrease in *Ufm1* or *Ufbp1* mRNA levels compared to siControl-treated cells; the efficiency of knock-down did not increase after 72 hours of incubation (data not shown). Insulin release in these silenced cells was not affected compared to the control cells (Figure S5), indicating that neither UFM1 nor UFBP1 are required for glucose regulated insulin secretion.

### ER stress-induced apoptosis is increased after *Ufm1*, *Ufbp1* and *Ufl1* knockdown

**Figure 5:**
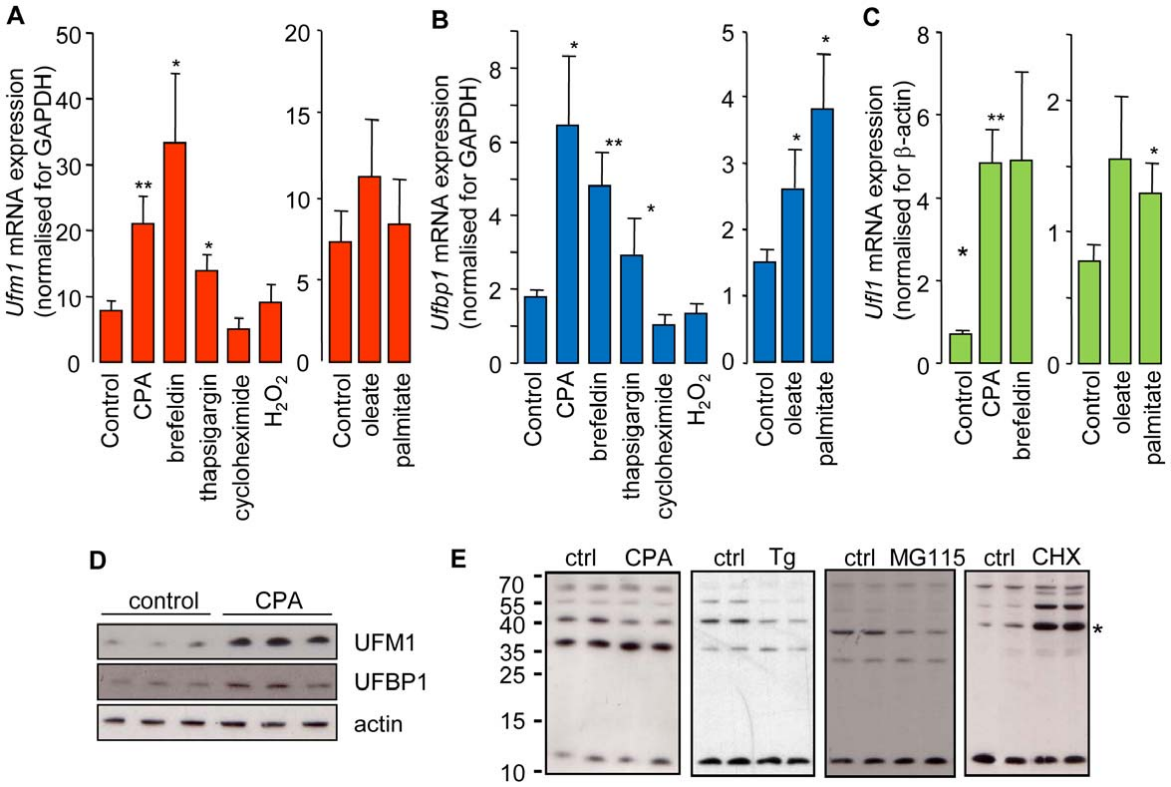
*Ufm1*, *Ufbp1*and *Ufl1* expressionis induced upon ER stress. A mRNA expression level of *Ufm1* (A), *Ufbp1*(B) or *Ufl1* (C) after exposure of INS-1E cells to 25 µM CPA, 1 µM thapsigargin, 1 µg/ml brefeldin A, 5 µg/ml cycloheximide, 30 µM H_2_O_2_, 0.5 mMoleate and 0.5 mMpalmitate for 14 hours, measured via qPCR and normalized for GAPDH (*Ufm1* and *Ufbp1*) or actin (*Ufl1*). Data are means±SEM, n = 4–6, *, p≤0.05; **, p≤0.01, paired student t-test, D UFM1 and UFBP1 protein expression is induced in cells exposed for 14 hours to CPA. Actin is shown as a control for protein loading, E UFM1-UFBP1 conjugates. Incubation of MIN6 or INS1 cells with ER stressors (25 µM CPA, 14h, INS1 cells; 1 µM thapsigargin (Tg), 1h, MIN6 cells) or proteasome inhibitor (100 µM MG115, 2h, INS1 cells) decreased conjugation, while incubation with a translational inhibitor (10 mg/l cycloheximide (CHX), 2h, MIN6 cells) increased the conjugate formation. * = UFM1−UFBP1 conjugate, 10 kDa = free UFM1.

Since UFM1 and UFBP1 are co-localized in the ER and a possible effect of ER stress on *Ufm1* expression was suggested [31], [32], we analyzed the role of UFM1 and UFBP1 during ER stress. A 14-hour exposure of INS-1E cells to cyclopiazonic acid (CPA), a potent ER Ca^2+^ ATPase pump inhibitor and pharmacological inducer of ER stress, markedly induced *Ufm1* and *Ufbp1* mRNA expression (Figure 5A,B). This induction was confirmed at the protein level (Figure 5D). The chemical ER stressors thapsigargin (another inhibitor of the ER Ca^2+^ ATPase pump) and brefeldin A (an inhibitor of ER-to-Golgi transport) also induced *Ufm1* and *Ufbp1*mRNA , while cyclohexamide and H_2_O_2_, two non-ER stressors, had no influence on the expression levels(Figure 5A,B), suggesting that ER stress mediates the upregulation. Free fatty acids (FFAs) are physiologically more relevant ER stress inducers in beta cells [33], [34], [35]. Exposure of INS-1E cells to the FFAs oleate or palmitate for 14 hours did not increase *Ufm1* expression (Figure 5A), but *Ufbp1* was clearly induced (Figure 5B). Also the expression of *Ufl1*, the E3 enzyme of UFM1, was increased after ER stress, similar to *Ufm1* and *Ufbp1* expression (Figure 5C).

The effect of ER stressors on UFM1 conjugation was also analyzed. Figure 5E shows a clear reduction of UFM1-UFBP1 conjugation after ER stress. The reduction in conjugation was not a consequence of increased proteasome activity since the conjugates also decreased after treatment of the cells with MG115, a potent proteasome inhibitor (Figure 5E). In contrast, decreasing the ER load via translational inhibition with cycloheximide resulted in an increase in UFM1-UFBP1 conjugation. These data suggest that UFM1-UFBP1 conjugation depends on the protein load in the ER, high when protein load is low and low when protein load is high.

**Figure 6:**
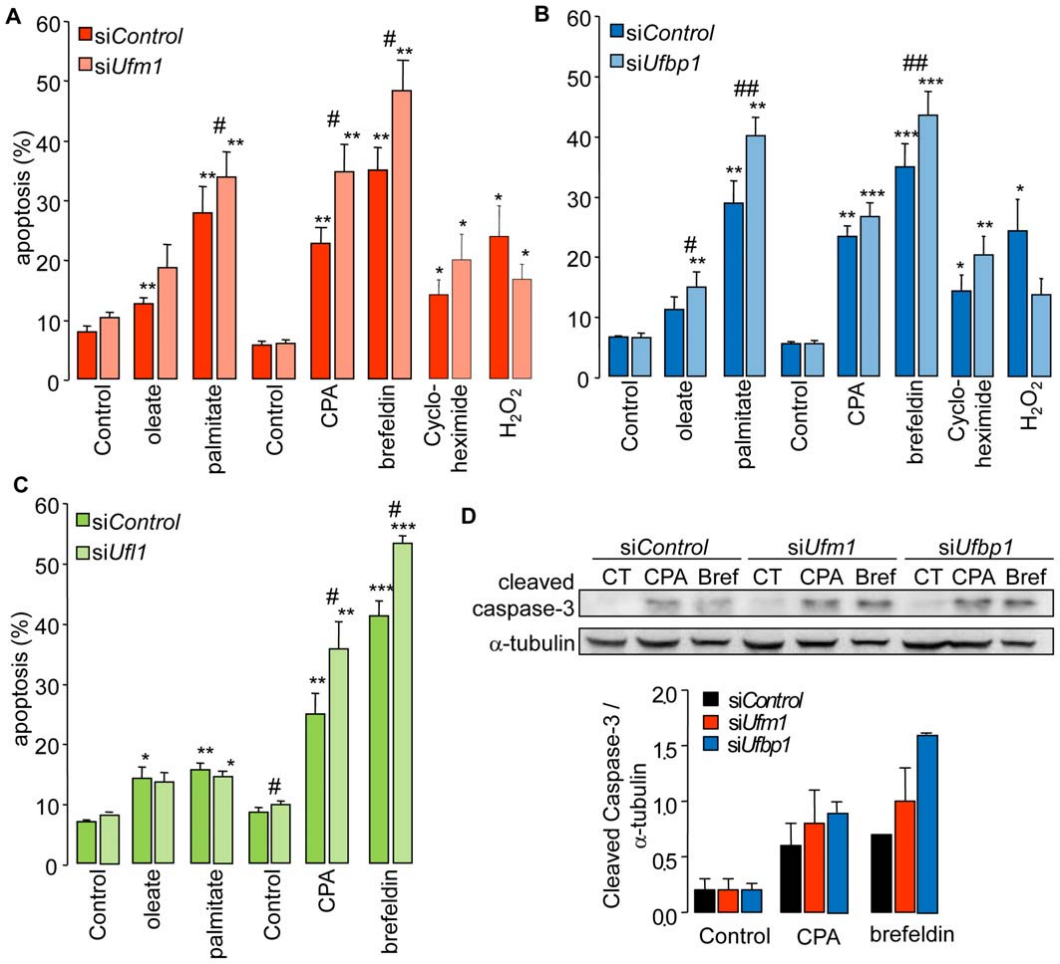
UFM1 and UFBP1 are involved in ER stress induced apoptosis. INS-1E cells were treated with the ER stressors oleate, palmitate, cyclopiazonic acid (CPA) and brefeldin A or the ER stress-independent apoptosis inducers cycloheximide and H_2_O_2_, and silenced for*Ufm1* (A), *Ufbp1* (B) or *Ufl1* (C). Apoptosis was evaluated by Hoechst/PI staining. Data are means±SEM of 3–7 independent experiments. Paired student *t* test: *, p≤0.05; **, p≤0.01; ***, p≤0.001 compared to control treatment; #, p≤0.05; ##, p≤0.01 silenced cells compared to siControl cells, D Caspase 3 activation in *Ufm1* and *Ufbp1* silenced cells after treatment with the ER stressors CPA and brefeldin A (Bref). The densitometric quantification of the immunoblots is shown in the lower panel. Cleaved Caspase-3 signal was normalized for α-tubulin expression. The results are means ± SEM of 2–3 independent experiments. In the assays, the respective controls contain the vehicles ethanol and DMSO.

Because apoptosis is triggered when the ER stress response fails to restore ER homeostasis, we analyzed the effect of a reduced *Ufm1*and*Ufbp1* expression on beta cell survival. We analyzed also the effect of *Ufl1* knockdown on apoptosis, to be able to investigate the importance of the conjugation between UFM1 and UFBP1, since a reduced UFL1 expression results in a significant reduction of UFM1-UFBP1 conjugation [1]. The knockdown efficiency during the whole experiment is shown in Figure S6.*Ufm1*silencing had no effect on basal apoptosis, but significantly increased apoptosis upon ER stress induced by palmitate, CPA and brefeldin A compared to siControl cells (Figure 6A). *Ufbp1* silencing increased oleate-, palmitate- and brefeldin A-induced apoptosis (Figure 6B) and *Ufl1* silencing increased CPA- and brefeldin A- induced apoptosis by 30–40% compared to siControl treated cells (Figure 6C). *Ufm1* and *Ufbp1* silencing had no affect on apoptosis induced by non-ER stressors such as cycloheximide- or H_2_O_2_ (Figure 6A, B), indicating that these proteins specifically act on ER stress-induced apoptosis. The sensitization of beta cells to ER stress-induced apoptosis was confirmed using a second method, namely caspase 3 cleavage. The knockdown of *Ufm1* and *Ufbp1* lead to enhanced caspase activation by CPA and brefeldin A (Figure 6D). Although *Ufbp1* and *Ufm1* silencing sensitized beta cells to ER stress, the expression of *BiP*, *Chop* or spliced *Xbp1* was not altered (Figure S7).

These data show that *Ufm1* and *Ufbp1* expression is upregulated upon ER stress, while UFM1-UFBP1 conjugation is decreased under these conditions; furthermore, UFM1 and UFPB1 are needed to prevent beta cell apoptosis caused by accumulation of unfolded protein in the ER.

## Discussion

In this study we provide evidence for a role of UFM1 and UFBP1in ER stress-induced beta cell apoptosis. We also identified a second interacting protein of UFM1, namely CDK5RAP3/LZAP.

Eight UFM1 interacting proteins were isolated and identified via affinity purification and mass spectrometry (UFC1, UFBP1, UBA5, CDK5RAP3, HSPA8, BiP, UFL1 and PC). For two of these, UFBP1 and CDK5RAP3, the interaction could be confirmed *in vitro* by GST pull down. Two expected UFM1 interacting proteins (UBA5 and UFC1, the known activating and conjugating enzymes of human UFM1 [4]) were identified. However, each of these two proteins was identified ashaving two distinct molecular masses. This could be explained by the fact that during denaturation of the protein extracts, the interaction between UFM1 and UBA5 or UFC1 was partially sustained. The screen also identified pyruvate carboxylase, but this is likely to be an artifact since the STrEP-tag affinity purification is based on streptavidin-biotin binding and pyruvate carboxylase has biotin as a natural co-factor. Moreover, pyruvate carboxylase protein is very abundant in beta cells [25]. Interestingly, the important ER chaperoneBiPwhich is known to play an important role in the unfolded protein response of protein secreting cells [36] and in particular for pro-insulin folding in the pancreatic beta cell [22], [37] interacted with UFM1 in our screen.While we could not confirm this interaction in GST pull down experiments and co-immunoprecipitations, we found an interaction between UFBP1 and BiP after co-immunoprecipitation.

In a large scale mapping of human protein-protein interactions by immunoprecipitation and mass spectrometry, Ewing et al. provided evidence for an interaction of CDK5RAP3 with UFM1, UFC1, UFL1 and UFBP1 [38]. Since we found an interaction of mouse UFM1 with the same proteins, this suggests that a similar complex exists both in mouse and human. Although we could not confirm the interaction between UFM1 and UFL1 via GST pull downs *in vitro*, these data, together with our finding that UFL1 interacts*in vitro* to CDK5RAP3, suggest that UFL1 is part of the complex, but that it has no direct interaction with UFM1. Tatsumi et al. reported that UFL1 is an E3 ligase for UFM1, although it has no structural characteristics of the typical E3 enzymes [1]. They found an interaction between UFM1-UFL1, UFL1-UFC1 and UFM1-C20orf116, using immunoprecipitations. Since under these conditions, also all other proteins (e.g. CDK5RAP3) are present, it is possible that the interaction between UFM1 and UFL1 is indirect, as shown in our experiments. Another possibility is that under our GST-pull down conditions, the required environment for interaction was not created, although we do see an interaction between UFL1 and CDK5RAP3 under these conditions. An interaction between UFL1 and CDK5RAP3 was recently also shown by some other groups [26], [27], [28]. The covalent binding between UFM1 and UFBP1 is supported by the MS/MS identification of UFBP1 in a protein fragment which is about 10 kDa higher than the predicted molecular weight of UFBP1, and which contains a modified lysine 268. The increase in molecular weight is in perfect agreement with a UFM1 modification (∼9.1 kDa). Also the presence of a UFM1 conjugate at the same height and with the same cellular localization as UFBP1 supports the covalent binding.

Detailed analysis of the protein sequence of UFBP1 revealed the presence of a signal peptide, a nuclear localization signal and a PCI domain [29]. Based on the presence of this signal peptide, UFBP1 is predicted to play a role in the secretory pathway (Target P1.1 prediction, [39]). Deletion of the signal peptide did not prevent the binding between UFM1 and UFBP1 *in vitro* but it prevented the ER localization of both UFBP1 and UFM1. Both proteins were mainly localized in the nucleus when the signal peptide was deleted, which could be explained by the presence of a nuclear localization signal in UFBP1. The function of this nuclear localization signal seemed to be overruled by the signal peptide, since the full length protein is localized in the ER. UFBP1 also contains a PCI domain, an alpha-helical domain of about 200 residues, which is generally localized at the C-terminus of the protein [30]. The COP9 signalosome is a conserved eight-subunit complex, which can physically associate with the 26S proteasome, and may function as an alternate lid for the proteasome [40]. A PCI domain can serve as a structural scaffold for multi-protein complexes or proteasome regulators. However, deletion of the PCI domain resulted in a stronger interaction between UFM1 and UFBP1, compared to the interaction with the full length UFBP1. Furthermore, UFBP1^1–219^ co-localized even stronger with UFM1 to the ER than UFBP1 did. We analyzed several conserved lysine residues (K121/K122, K192/K194 and K159) outside the signal peptide and PCI-domain via GST pull down, but none of these lysine residues seemed to play a role on their own for interaction with UFM1 (results not shown).K268 may not be the only lysine residue involved in UFM1 conjugation since the mutants UFBP1^K268R^ and UFBP1^1–219^ are still able to form UFM1 conjugates. Our interpretation is that while K268 is the main lysine residue for conjugation, but when this residue is deleted, other lysine residues can take over. Most likely, UFM1 can interact with UFBP1, both covalent and non-covalent, since the mutants UFM1^G83A^, UFBP1^K268R^ and UFBP1^1–219^ can still co-localize in the ER. However, we cannot exclude that endogenous UFM1 and UFBP1 play a role in this co-localization (e.g. via dimerization). Another possibility is that overexpression of UFBP1 caused changes in the ER (*e.g.* massive amplification of ER network as recently reported [1]), and that these changes had an influence on the localization of UFM1.

Since UFBP1 was predicted to function in the secretory pathway and UFM1 is mainly expressed in secretory cells, we analyzed the effect of *Ufm1* and *Ufbp1* silencing on the glucose stimulated insulin release. However, no clear effect of decreased UFM1 and UFBP1 protein levels on insulin release was observed. Also no secreted UFBP1 could be detected in the medium (results not shown).

We showed that *Ufm1*, *Ufbp1*and *Ufl1* expression in INS1 cells was increased upon ER stress induced by chemical ER stressors and by the FFAs palmitate and oleate, fitting with other data [31], [32]. The protein synthesis/folding load in the ER had a strong influence on the amount of detected UFM1-UFBP1 conjugates: increasing the protein load with ER stressors (cyclopiazonic acid, thapsigargin) decreased the abundance of conjugates, while a decrease in protein load with the translational inhibitor cycloheximide increased UFM1-UFBP1 conjugation. One possible interpretation is that UFM1-UFBP1 conjugates are used during protein folding in order to prevent ER stress and beta cell apoptosis. A difference in conjugate stability under different conditions could be the reason why after cycloheximide treatment the amount of conjugates increased, while free UFM1 protein and *Ufm1* mRNA levels stayed the same. Apoptosis induced by non-ER stressors was not influenced by the knockdown of UFM1 or UFBP1. The fact that *Ufl1* knockdown also enhanced apoptosis, similar to *Ufm1* and *Ufbp1* silenced cells, suggests that it is the conjugation between UFM1 and UFBP1 that is required, rather than the expression of the proteins itself. The exact mechanism how UFM1-UFBP1 conjugation can protect the cell is not known. Although *Ufbp1* and *Ufm1* silencing sensitized beta cells to ER stress, the expression of *BiP*, *Chop* or spliced *Xbp1* was not altered (Figure S7). However, we did see an impairment of ERAD activity upon *Ufbp1* knockdown (Figure S8).

Importantly, in a recent study, Lu et al. identified UFM1 as a potential factor associated with the development of type 2 diabetes [41]. UFM1 expression was 1.94 (protein) and 1.53 (mRNA) times higher in MKR mice (Type 2 diabetes model) than in wild type mice. MKR islets also contain more molecular chaperones (GRP78 and GRP94) and proteins involved in ERAD [42]. In light of our present data, this increased UFM1 expression may be the result of ER stress in beta cells *in vivo*, and might be part of a protective response.

CDK5RAP3/LZAP is a putative tumor suppressor, as it was shown to activate the tumor suppressor p53 and to inhibit growth of tumor cell lines *in vitro* [43]. Furthermore, CDK5RAP3 promotes apoptosis in response to genotoxic agents [44] and is an inhibitor of NF-κB [45]. Very recently, Shiwaku et al. demonstrated that UFL1 (Maxer) is also anchored in the ER and interacts with its N-terminal cytosolic part to CDK5RAP3. With this interaction, UFL1 prevents CDK5RAP3 to inhibit cyclin D1 expression, required for G1/S transition. Further experiments are necessary to clarify the role of the interaction between UFM1 and CDK5RAP3 in the cell.

In summary our study shows that UFM1-UFBP1interaction occurs primarily in protein secreting cells like pancreatic beta cells; in such cells this interaction may protect the cells against ER stress and apoptosis.

## Materials and Methods

### Tissue isolation, cell culture and transient transfection

All procedures involving mouse tissues were conducted according to protocols and guidelines approved by the K.U. Leuven animal welfare committee (ID 085/2003).Mouse tissues were isolated from male C57Bl/6J mice, between 12 and 15 weeks old. Islets were isolated by injection of collagenase P (Roche) in the pancreatic duct followed by 3 min digestion at 37°C. Islets were hand-picked in HEPES Krebs buffer (20 mM HEPES, pH 7.4; 119 mMNaCl; 4.75 mMKCl; 2.54 mM CaCl_2_; 1.2 mM MgSO_4_; 1.18 mM KH_2_PO_4_; 5 mM NaHCO_3_) containing 5 mM glucose, and used directly for RNA or protein isolation. The mouse insulin-producing MIN6 cell line (p. 20–30) was kindly donated by Dr. E. Yamato (Osaka University, Japan) [46] and cultured in DMEM (Invitrogen) (25 mM glucose) equilibrated with 5% CO_2_ and 95% air at 37°C. The medium was supplemented with 15% decomplemented fetal calf serum(FCS, Invitrogen), 70 µMβ-mercaptoethanol, 4 mMglutaMAX, 50 U/ml penicillin and 50 µg/mlstreptomycin. The rat insulin-producing INS1-832/13 cell line (p. 50–70) [47] was cultured in RPMI 1640 medium supplemented with 10% decomplemented FCS, 1 mM sodium pyruvate, 50 µM β-mercaptoethanol, 10 mM HEPES, 100 U/ml penicillin and 100 µg/ml streptomycin. The rat insulin-producing INS-1E cell line (p. 55–75) (a kind gift from Dr. C. Wollheim, Centre Medical Universitaire, Geneva, Switzerland) was cultured in RPMI 1640 (with GlutaMAX-I) containing 5% FCS, 10 mM HEPES, 1 mM sodium pyruvate, 100U/ml penicillin, 100 µg/ml streptomycin and 50 µM 2-mercaptoethanol [48]. The mouse glucagon-producing αTC1-6 cell line [49] was cultured in DMEM/F12 medium supplemented with 10% decomplemented FCS, 25 mM glucose, 50 U/ml penicillin and 50 µg/ml streptomycin. Treatment of INS1 cells with oleate and palmitate (sodium salt, Sigma), cyclopiazonic acid (CPA, 25 µM, Sigma), thapsigargin (1 µM, Sigma), brefeldin A (1 µg/ml, Sigma), cycloheximide (5 µg/ml) or H_2_O_2_ (30 µM)were performed as described before [50]. For the free fatty acid (FFA) treatment, medium was used containing 1% FCS and 1% charcoal-absorbed BSA. FFAs were dissolved in 90% ethanol and diluted 1∶100 to a final concentration of 0.5 mM, corresponding to a FFA/BSA ratio of 3.4 [50], [51].To suppress Ufm1 expression in 832/13 cells, 25 nM of a Ufm1-specific siRNA was transfected, using Dharmafect 1 (Dharmacon) or Lipofectamine 2000 (Invitrogen), according to the manufacturer's protocol. Control cells were treated with an siRNA with no known sequence homology (siControl), as previously described [52]. For overexpression experiments in MIN6 cells, AMAXA technology was used. Briefly, 10^7^ cells were electroporated with 5 µg pDNA, using the T20 program, and recovered for 1 day in RPMI medium. For overexpression in INS1-832/13 cells, 800K cells in a 6-well were transfected with Fugene HD (Roche, Switzerland), using a 2 µg/8 µl pDNA/fugene HD ratio.

### RNA isolation and quantitative RT-PCR

*Total RNA* from mouse tissues was extracted using Trizol reagent according to the manufacturer's protocol (Invitrogen), followed by a cleanup procedure with RNeasy columns (Qiagen). RNA from mouse islets as well as mouse pituitary and adrenal gland was extracted using the Absolutely RNA microprep (Stratagene). For MIN6 and INS1-832/13 cells, we used the PureLink micro-to-midi RNA kit (Invitrogen). The total RNA quantity and quality was determined using the NanoDrop ND-1000 spectrophotometer (NanoDropTechnologies) and the 2100 Bioanalyzer (Agilent, Germany), respectively. Total RNA profiles of all tested samples were similar with sharp 18S and 28S rRNA peaks on a flat baseline. Poly(A)^+^-RNA was isolated from INS-1E cells as described [34]. *For microarray*, total cellular mRNA (2 µg, except for islets, adrenal gland and pituitary where 1 µg was used) was reverse transcribed into cDNA (SuperScript Choice System, lnvitrogen, using oligo-dT primers and a T7 RNA polymerase promoter site). In all cases the cDNA was *in vitro* transcribed and biotin-labeled for microarray analysis using a commercially available kit (Affymetrix IVT labeling kit, CA). The concentration of labelledcRNA was measured using the NanoDrop ND-1000 spectrophotometer. Labeled cRNA was fragmented in a fragmentation buffer during 35 min at 94°C. The quality of labeled and fragmented cRNA was analyzed using the Agilent bioanalyzer 2100. Fragmented cRNA was hybridized to the mouse 430 2.0 (Affymetrix) array during 16 h at 45°C. Washing and staining of the arrays was performed in a fluidics station (Affymetrix) and afterwards scanned with the Affymetrix 3000 GeneScanner. All image files were analyzed using GCOS with the MAS 5 algorithm. The fluorescence intensity of each individual chip was scaled to a target intensity of 150 using the global scaling method. All quality controls of the arrays were according to manufacturer's criteria. All data files have been deposited in the NCBI Gene Expression Omnibus (GEO, http://www.ncbi.nlm.nih.gov/geo/), are MIAME compliantand are accessible through GEO series accession number GSE24207.

*For quantitative RT-PCR*, 1 µg total RNA was reversed transcribed with the RevertAid H Minus First strand cDNA synthesis kit (Fermentas) or using GeneAmp RNA PCR (Roche). Targets were amplified from 5 ngcDNA in a rotor-gene 3000 (Corbett Research) with absolute QPCR mix (ABgene) or on a LightCycler (Roche) with SYBR Green PCR master mix (Qiagen) using the following oligonucleotide sequences: forward Mm_*Ufm1*, 5′-GGTGTGTGTCAGGCGGTTC-3′; reverse Mm_*Ufm1*, 5′-CATTCCCAGCAGTCTGTGCAG-3′; probe Mm_*Ufm1*, 5′-(6-FAM)ACGTTGACGTCGGACCCGCGGC(TAMRA)-3′; forward Rn_*Ufm1*, 5′-GGTTTGAGTACCAGGCGGTTC-3′; reverse Rn_*Ufm1*, 5′-CGTTTCCAGCAGTCTGTGCAG-3′; probe Rn_*Ufm1*, 5′-(6-FAM) ACGCTCACGTCGGACCCGCGGC(TAMRA)-3′, forward beta-actin, 5′-AGCCATGTACGTAGCCATCCA-3′; reverse beta-actin, 5′-TCTCCGGAGTCCATCACAATG-3′; probe beta-actin 5′-(6-FAM) TGTCCCTGTATGCCTCTGGTCGTAC(BHQ1)-3′; forward Rn_*Ufbp1*, 5′- GGAAGAAGTGGATGAGAACGAGG-3′; reverse Rn_*Ufbp1*, 5′- CCTGTTGGGTGAACTTCTGC-3′; probe Rn_*Ufbp1* 5′-(6-FAM) AGCTGCTGTTCCAGCCCAGGAGGAAGAAG(TAMRA)-3′, forward Rn_Ufl1, 5′-CAAGGACTTACTTACAAGAAGAGGTTTC-3′; reverse Rn_Ufl1, 5′-GGTACACACTGTCTTCAGC-3′; probe Rn_Ufl1, 5′(6-FAM)CAGATGACACACAGACTGCTCTGACCAAGC(TAMRA)-3′. Primers for glyceraldehyde-3-phosphate dehydrogenase (GAPDH), *Chop,BiP* and spliced *XBP1* have been reported before [34], [53].

### Plasmid construction and siRNA

Mouse *Ufm1* was amplified by PCR using primers 5′-GTGCATATGTCGAAGGTGTCCTT-3′ and 5′-GAGGGATCCTTAGCAGCTTCCAACTCG-3′ for cloning in pET16 (*Nde*l/*Bam*HI), 5′CGTAAGCTTCCATGTCGAAGGTGTCCTT-3′ and 5′-GCGAATTCTATTAGCAGCTTCCAACTCG-3′ for cloning in pcDNA3, eGFP(C1) and pmRFP(C1) (Invitrogen) (*Hind*IIl/*Eco*RI), 5′-CCGAATTCCATGTCGAAGGTGTCCTT-3′ and 5′-GAGGGATCCTTAGCAGCTTCCAACTCG-3′ for cloning in pEXPR-IBA105 (Westburg) (*Eco*RI/*Bam*HI), 5′-CGGGATCCATGTCGAAGGTGTCCTTTAA-3′ and 5′-GAGGGATCCTTATCCAACTCGGTCTCTAGG-3′ for cloning in pGEX-2TK (*Bam*HI).*Ufm1*(G83A) was obtained via site-directed mutagenesis (Stratagene).*Ufbp1* was amplified via PCR using primers 5′-TGATCTAGAATGGTGGGGCCCTGGGTGTATC-3′ and 5′-GACCTCGAGGGCTGAAGCCTGGGCAGGGAG-3′ for cloning in pEXPR-IBA103 (Westburg) *(Xba*I/*Xho*l).The PCI domain of *Ufbp1* was removed by cloning of the *Xba*l/*Eco*RI fragment of plasmid pEXPR-IBA103_*Ufbp1*_STrEP in the (*Xho*l/*Eco*RI), resulting in the plasmid pEXPR-IBA103_*Ufbp1* ^1–219^_STrEP. An eGFP tag was cloned in this construct via *Xho*l/*AfI*ll cloning, or removed by *Xho*l/*Afl*Il digestion and Klenow fill in reaction. Ufbp1 without a signal peptide (*Ufbp1* ^29–314^) was generated via PCR using primers 5′-TGATCTAGAATGGCAGCAGCTGACGGAGAACC-3′ and 5′-GACCTCGAGGGCTGAAGCCTGGGCAGGGAG-3′ for cloning in the pEXPR-IBA103 vector (*Xba*l/*Xho*l).The STrEP-tag of this construct was replaced by eGFP via *Xho*l/*Afl*ll cloning, or removed via *Xho*l/*Afl*Ildigestion and Klenow fill in reaction. The lysine mutants of *Ufbp1* were obtained via site-directed mutagenesis (Stratagene). Mouse *Ufl1* was amplified via PCR using primers 5′-TGAAAGCTTCAATGGCGGACGCCTGGGAGG-3′ and 5′-GTAGAATTCTTATGCTCCTCTGTGACAGATGATTTCC-3′, and cloned in pcDNA3 (*Hind*III/*Eco*RI),mouse *Cdk5Rap3* using primers 5′-TGAAAGCTTCAATGCAGGACCATCAGCACG-3′ and 5′-GTAGAATTCTCACAGGACGGCCACTGTATCTC3′, and cloned in pcDNA3 (*Hind*III/*Eco*RI). *Cdk5Rap3* (*Hind*III/Klenow/*EcoR*I) from this construct was cloned in pGEX-2TK (*Sma*I/*EcoR*I). Mouse *BiP* was amplified using primers 5′-TGAAAGCTTCAATGATGAAGTTCACTGTGG-3′ and 5′-GACCTGCAGACAACTCATCTTTTTCTGATGTATCC-3′, and cloned in pcDNA3 (*Hind*III/*Pst*I).The plasmid pCI-NeoHA-CD3delta was a kind gift of Dr. A. Weissman [54].

*ON*TARGETplus*Ufm1*siRNA (Dharmacon) against the rat *Ufm1* sequence 5′-GUUUGCAGAAGAGUUUAA-3′ or 5′-GCUACAAGUGCGAUUAUUAUU-3′,a pool of four siRNA oligonucleotides (5′-CCUUUGUGGUAGAAGAAGA-3′, 5′-GGGCAAGUUCAUCUACAUA-3′, 5′-GGGUGAAGCUGCUGUUCCA-3′, 5′-GCGAGUGACCUGGGAAGAA-3′) targeting rat *Ufbp1* (Dharmacon) or (5′-AGUAAACAUUGUCGACUUAUU-3′, 5′-GAACAUGGGUUGACGUUUCUU-3′, 5′-UGUUGUGGUCAGCGAGAAAUU-3′, 5′-AAGACAGUGUGUACCGAUAUU-3′) targeting rat *Ufl1* were used. A non-targetingsiRNA or pool was used as negative control (Dharmacon or Qiagen).

### Antibodies

Polyclonal UFM1 and UFBP1 specific antibodies were raised against the recombinant His-tagged UFM1 and the UFBP1 peptide C-RKRLESQREAEWKKE (synthesised by EZbiolab), respectively. The UFBP1 peptide was conjugated with maleimide-activated mcKLH following the manufacturer's protocol (Pierce). Rabbits were immunized with antigen (His_UFM1 or UFBP1 peptide_KLH) in Freund's complete adjuvant and boosted after 14 days using Freund's incomplete adjuvant. Serum was affinity purified using CNBr-activated sepharose 4 fast flow beads (Amersham) linked with recombinant His-UFM1 or UFBP1 peptide conjugated to BSA. GST antibody was from Santa Cruz, BiP, β-actin and GAPDH antibody from Abcam, HSPA8 antibody from Gentaur, LAMP2 and HA antibody from Sigma and GDH antibody was a kind gift from M. Franssen (K.U. Leuven, Belgium). BiP and UFBP1 antibodies were conjugated to HRP using the ‘lightning-link HRP conjugating’ kit (Innova Biosciences) for detection after immunoprecipitation.

### Protein isolation, subcellular fractionation and immunoprecipitation

For total protein extraction, tissues were immediately washed with PBS after dissection and lysed in S1 buffer (50 mMTris, pH 8; 0.4% NP-40; 150 mMNaCl; 1 mM EDTA; proteinase inhibitor cocktail tablet (Roche); 1 mM PMSF; 2 mM N-ethylmaleimide), using a pestle for homogenization. Protein concentrations were measured via Dc protein assay (Bio-Rad). For subcellular fractionation, MIN6 cells were homogenised in freshly prepared HMB buffer (250 mM sucrose; 5 mM MOPS, pH 7.2; 1 mM EDTA; 1 mM DTT; 1 mM PMSF; 2 mM N-ethylmaleimide; proteinase inhibitor cocktail tablet) with a metal douncer (20 strokes). The homogenate was centrifuged at 770×g for 10 min. The pellet (N) was resuspended in 1 ml HM buffer. The supernatant was centrifuged for an additional 10 min at 2330×g. The pellet (M) was again resuspended in 1 ml HMB buffer, and the supernatant centrifuged at 13000×g for 20 min. The resulting pellet (L) was resuspended in 1 ml HM buffer and the supernatant centrifuged at 100000×g for 60 min. After resolving the pellet (P) in 1 ml HM buffer and bringing the supernatant (S) volume to 1 ml, all the protein fractions were precipitated with 7% TCA and 0.015% deoxycholate. The pellets were resolved in SDS sample buffer. Protein extracts were separated by 4–12% SDS-PAGE (Invitrogen).For co-immunoprecipitation, cell lysates were incubated with BiP, UFBP1 or UFM1 antibody and bound to protein A-TSK sepharose (Affiland). After elution, proteins were separated by SDS-PAGE and detected via immunostaining.

### GST protein isolation, in vitro transcription/translation and GST pull down assay

*GST protein isolation. E. coli* pLYS cells were used to produce GST and GST-Ufm1 recombinant protein. 1 mM IPTG was used to induce protein expression at 30°C for 2 hours. Bacterial cells were resuspended in lysis buffer (50 mMTris, pH 7.5; 0.45 M NaCl; 0.1% Triton-X-100; 1 mM DTT; 0.1% β-mercaptoethanol) and disrupted by sonication (6×10″). After centrifugation (12000×*g*, 20 min), the protein extracts were immobilized on glutathione-agarose beads (Sigma) for 1 hour at 4°C. The beads were then washed with washing buffer (50 mMTris, pH 7.5; 0.15 M NaCl; 0.1% Triton-X-100; 1 mM DTT; 0.1% β-mercaptoethanol). The GST fusion proteins were eluted from the beads with reduced glutathione (Acros) in 100 mMTris, pH 7.5, and after concentration, dialysed overnight in PBS +500 mMNaCl.*In vitro transcription/translation*. The TnT T7 transcription/translation kit (Promega), using rabbit reticulocyte lysates, was used to prepare *in vitro* ^35^S-methionine labeled target proteins, following the manufacturer's protocol.*GST pull-down assay*. 100 µl glutathione-agarose beads were blocked in Tris-buffer (50 mMTris, pH 7.5; 100 mMNaCl)+1 mg/ml BSA and 0.5% Triton-X-100 for 15 min at 4°C. After washing and resuspending the beads with binding buffer (50 mMTris, pH 7.5; 100 mMNaCl, 0.1% NP40; 1 mM DTT), they were incubated with 550 pmol GST protein or GST-Ufm1 protein for 30 min at 4°C. The beads were then washed 2 times with binding buffer and resuspended in 50 µl binding buffer. MIN6 cell-lysates and ^35^S-labelled proteins (40 µl) were pre-cleared on GST coupled beads for 30 min at 4°C. 40 µl coupled beads were incubated with 10 µl pre-cleared MIN6 lysates (25×10^3^ cells) and 1/3 pre-cleared ^35^S-labelled protein in ubiquitilation buffer (50 mMTris, pH 7.5; 100 mMNaCl, 0.1% NP40; 1 mM DTT; 2 mM ATP; 5 mM MgCl_2_) (final volume, 200 µl) for 30 min at 40°C. The beads were then washed 3 times with binding buffer. Bound proteins were released from the beads by boiling in SDS sample buffer and separated on a 4–12% Tris-Glycine gel in MES buffer. The gel was dried and analyzed via phosphorimaging.

### Apoptosis

The percentage of viable, apoptotic, and necrotic cells was determined following staining of INS-1E cells with the DNA binding dyes propidium iodide and Hoechst 33342, as described [50]. For caspase 3 cleavage measurements, cells were washed with cold PBS and lysed with Laemmli buffer. Lysates were then resolved by 15% SDS-PAGE and transferred to a nitrocellulose membrane. Cleaved caspase-3 (Asp175, Cell Signaling, 1/1000) and α-tubulin (Sigma, (1/5000) were used as primary antibodies. Horseradish-peroxidase-labeled rabbit and mouse antibodies (Thermo Scientific) were used as secondary antibodies. Immunoreactive bands were revealed using the SuperSignal® West Femtochemiluminescent substrate (Thermo Scientific), detected using a LAS-3000 charge-coupled device camera and quantified with the Aida Analysis software (Fujifilm).

### Insulin release

Cells were treated with duplexes as described and grown to confluency. Insulin secretion was assayed as previously described [47]. Briefly, cells were washed with HEPES balanced salt solution (HBSS) (114 mMNaCl, 4.7 mMKCl, 1.2 mM KH_2_PO_4_, 1.16 mM MgSO_4_, 20mM HEPES, 2.5 mM CaCl_2_, 25.5 mM NaHCO_3_ and 0.2% BSA, pH7.2) with 3 mM glucose followed by a 2 hour pre-incubation in the same buffer. For glucose-stimulated insulin secretion, cells were incubated in HBSS for an additional 2 hours in the presence of 3 mM, 15 mM glucose or 15 mM glucose+30 mMKCl as indicated followed by collection of buffer for insulin radioimmunoassay (Coat-A-Count kit, DPC).

### STrEP-tag affinity purification and mass spectrometry

*STrEP-tag affinity purification*.150×10^6^ MIN6 cells were transfected with STrEP_*Ufm1*or *Ufbp1*_STrEP for 72 hours. After total protein extraction in S1 buffer, lysates were incubated with STrEP-Tactin beads (Westburg), following the manufacturer's protocol. The affinity purified proteins were separated on a 4–12% Tris/Glycine gel (Invitrogen) and stained with coomassie. The proteins that were present in the UFM1 purified samples and not in the control were picked for analysis via mass spectrometry.*Mass spectrometry*.Gel bands were picked in water, transferred to 100 µl fixation solution (50% methanol; 5% acetic acid) and rinsed three times with water and three times with ACN (LC-MS quality, chromasolv, Sigma). The gels were hydrated in 100 mM NH_4_HC0_3_, followed by dehydration in 100% ACN, each 10 min. This step was repeated twice prior to dehydrating the gel pieces in a speedvac. Gel pieces were rehydrated in digestion buffer (50 mM NH_4_HCO_3_; 5 mM CaCl_2_), containing 1 ng/µl modified trypsin (Promega) and incubated overnight at 37°C. The resulting peptides were extracted from gel in four steps: once with 50 mM NH_4_HCO_3_, twice with 50% ACN; 5% formic acid and once with 95% ACN; 5% formic acid, each 30 min. Supernatants were dried in a speedvac. Upon concentrating and desalting the tryptic fragments using Millipore C-18 ZipTips, the samples were mixed in a 1/1 v/v ratio with alpha-cyano-4-hydroxy-cinnamic acid matrix (saturated solution in 50% ACN; 2.5% TFA in HPLC water), spotted onto the MALDI target plate and allowed to air dry. MS/MS analysis was performed on a 4800 MALDI TOF/TOF (Applied Biosystems). The instrument was calibrated with the Applied Biosystems Calibration Mixture 1. Measurements were taken in the positive ion mode between 900 and 9000 m/z. Sequences were automatically acquired by scanning first in MS mode and selecting the 15 most intense ions for MS/MS using an exclusion list of peaks arising from trypticautodigestion. Data interpretation was carried out with the GPS Explorer software (V3.5) and database searching with the Mascot program (version 2.0.00). MS/MS searches were conducted with the following settings: MS/MS tolerance for precursor and fragment ions between 0.2 and 1 Da depending on the sample, methionine oxidation as variable modification and carbamidomethylation of cysteine as fixed modification. Trypsin was selected as enzyme and a maximum of one missed cleavage was allowed. Using these parameters the probability-based MOWSE score greater than the given cut-off value for MS/MS fragmentation data were taken as significant (p<0.05).

### Pulse/chase

Cells were pre-incubated for 1 hour in starving medium (RPMI 1640 without cystine and methionine (Sigma)) and then labeled with 200 µCi^35^S-methionine/cysteine (Perkin Elmer Easytag express protein labeling mix, specific activity 1175 Ci/mmol) for 1 hour at 37°C, followed by a 0, 30, 60 and 90 min chase at 37°C. The cell lysates were immunoprecipitated using HA antiserum (Sigma) and bound to protein A-TSK sepharose (Affiland). After elution, proteins were separated by SDS-PAGE and quantified for autoradiographic signals using ImageQuant software.

### Laser scanning confocal microscopy

Transfected INS1-832/13 cells were incubated with 1 µM ER-tracker blue-white DPX (Invitrogen), to stain the ER. Images were obtained with a Zeiss LSM510 laser scanning confocal microscope, using a 63× oil objective.

## Supporting Information

Figure S1 **mRNA expression of** ***Ufm1*** **in different mouse tissues, measured via QPCR and normalised for β-actin.** Data are means±SD, n≥3.
(TIF)

Figure S2 **Coomassie staining of the different fractions during STrEP-tag affinity purification of UFM1 (A) and UFBP1 (B).** Together with the eluate samples, a small aliquot was taken from the protein extract before and after binding to the beads and from the wash step. MIN6 cells were transfected with empty vector (E) or with a vector containing STrep-Ufm1 (U). Two different molecular weight markers were used. The protein fragments used for MS/MS identification are indicated and numbered. The identification of the proteins of UFM1 purification is shown in table 1, B Three distinct bands were shown to contain the UFBP1 protein. Fragment 1: the presence of both UFBP1 and G3P (glyceraldehyde 3-phosphate dehydrogenase, mass: 35810 Da) was demonstrated. The tryptic peptide containing the unmodified K268 (sequence: IQDLLTEGTLTGVIDGGK, mass: 2044 Da) in UFBP1 was measured with confidence (delta mass of 0.01 Da). Also the tryptic peptide following the K268 residue (sequence: FIYITPEELAAVANFIR, mass: 1967 Da) was demonstrated with confidence (delta mass: 0.04 Da), supporting the idea K268 is not modified and thereby excluded as a trypsin-cleaving site. Peptide fragmentation data was generated by MS/MS analysis and confirmed the peptide identities. Fragment 2: this band is identified as UFBP1. Again the 2044 (delta: 0.01 Da) and the 1967 (delta: 0.04 Da) masses were present. MS/MS analysis confirmed the AA sequences. The elution position in the SDS-PAGE gel corresponds to the expected molecular weight of the native protein (35956 Da). Fragment 3: Two proteins were identified: PDIA6 (protein disulfide-isomerase A6) and UFBP1. The position in the SDS-PAGE gel fits perfectly with the mass of PDIA6 (48070 Da) but is about 10 kDa too high for UFBP1. However, it is in perfect agreement with a UFM1 modification (+9.1 kDa) of UFBP1. Remarkably, both peptides (2044 and 1967), reporting the unmodified K268, are now missing, C Cellular fractionation of MIN6 cells overexpressing UFBP1_eGFP+UFM1 (left panel) or UFBP1K268R_eGFP+UFM1 (right panel). * = UFBP1−UFM1 conjugate, ← = UFBP1-eGFP−UFM1 conjugate.
(TIF)

Figure S3 **UFBP1 and UFM1 are co-localized in the ER.** A HeLa cells transfected or co-transfected with different eGFP or mRFP constructs as indicated on the picture, B INS1 cells co-transfected with mRFP-UFM1 and UFBP1-eGFP as depicted. Cells were also stained with an ER-tracker (blue). Pictures were taken with a 63× objective on a Zeiss confocal microscope.
(TIF)

Figure S4 **Cellular localization of UFM1 and UFBP1.** A INS1-832/13 cells transfected with wild type UFM1 or UFM1^G83A^, they both show similar localization. B INS1-832/13 cells co-transfected with UFM1 (WT or G83A-mutant) and UFBP1 (WT or K268R-mutant) as depicted on the picture. Cells were also stained with an ER-tracker (blue). Pictures were taken with a 63× objective on a Zeiss confocal microscope.
(TIF)

Figure S5 **Insulin secretion is not affected by Ufm1 or Ufbp1 silencing.** INS1-832/13 cells were silenced with Ufm1 (black bars) or Ufbp1 (grey bars) specific siRNA or with non-target siRNA (white bars). 48 hours after transfection, cells were incubated in medium with low (G3) or high (G15) glucose concentrations, or with high glucose concentrations together with 30 mMKCl. Data are means±SD, n = 4.
(TIF)

Figure S6 **Ufm1 and Ufbp1 mRNA expression during apoptosis experiment of** **Figure 5** **.** Expression was normalized to GAPDH (*Ufm1* and *Ufbp1*) or β-actin (*Ufl1*) expression. Data are means±SEM.
(TIF)

Figure S7 **ER stress markers are not enhanced after Ufm1 or Ufbp1 silencing.** INS-1E cells were transfected with siRNA against *Ufm1*, *Ufbp1* or non-target siRNA and treated with oleate, palmitate or CPA for 14 hours. mRNA expression of *BiP*, *Chop* and *Xbp1* splicing were analyzed using qPCR, and normalized for GAPDH. Data are means±SEM with n≥5, paired student t-test: *, p≤0.05; .**, p≤0.01; ***, p≤0.005.
(TIF)

Figure S8 **UFBP1 plays a role in ERAD.** \ERAD activity was analyzed by measuring CD3δ degradation in INS1-832/12 cells transfected with siRNA against *Ufm1*,*Ufbp1* and*Ufl1* and24 hours later with a CD3δ-HA expression construct, **A **Silencing of *Ufm1* and *Ufbp1* was analyzed via qPCR, **B **The transfected were starved for 1 hour and labeled with ^35^S-Met-Cys for 1 hour. After 0, 30, 60 and 90 min chase, cells were lysed and CD3δ-HA was immunoprecipitated with an HA antibody. After SDS-PAGE (upper panel), CD3δwas quantified (lower panel) and normalized for total ^35^S incorporation. Data are means±SEM, n = 6, *, p<0.01 with a Z-test on ratios of all time points comparing si*Ufbp1*vssiControl.
(TIF)
