# Supplementary figures and images for: Ubiquitin Fold Modifier 1 (UFM1) and Its Target UFBP1 Protect Pancreatic Beta Cells from ER Stress-Induced Apoptosis
Source: PLoS One. 2011 Apr 6;6(4):e18517. doi: 10.1371/journal.pone.0018517 (PMC3071830; doi:10.1371/journal.pone.0018517)

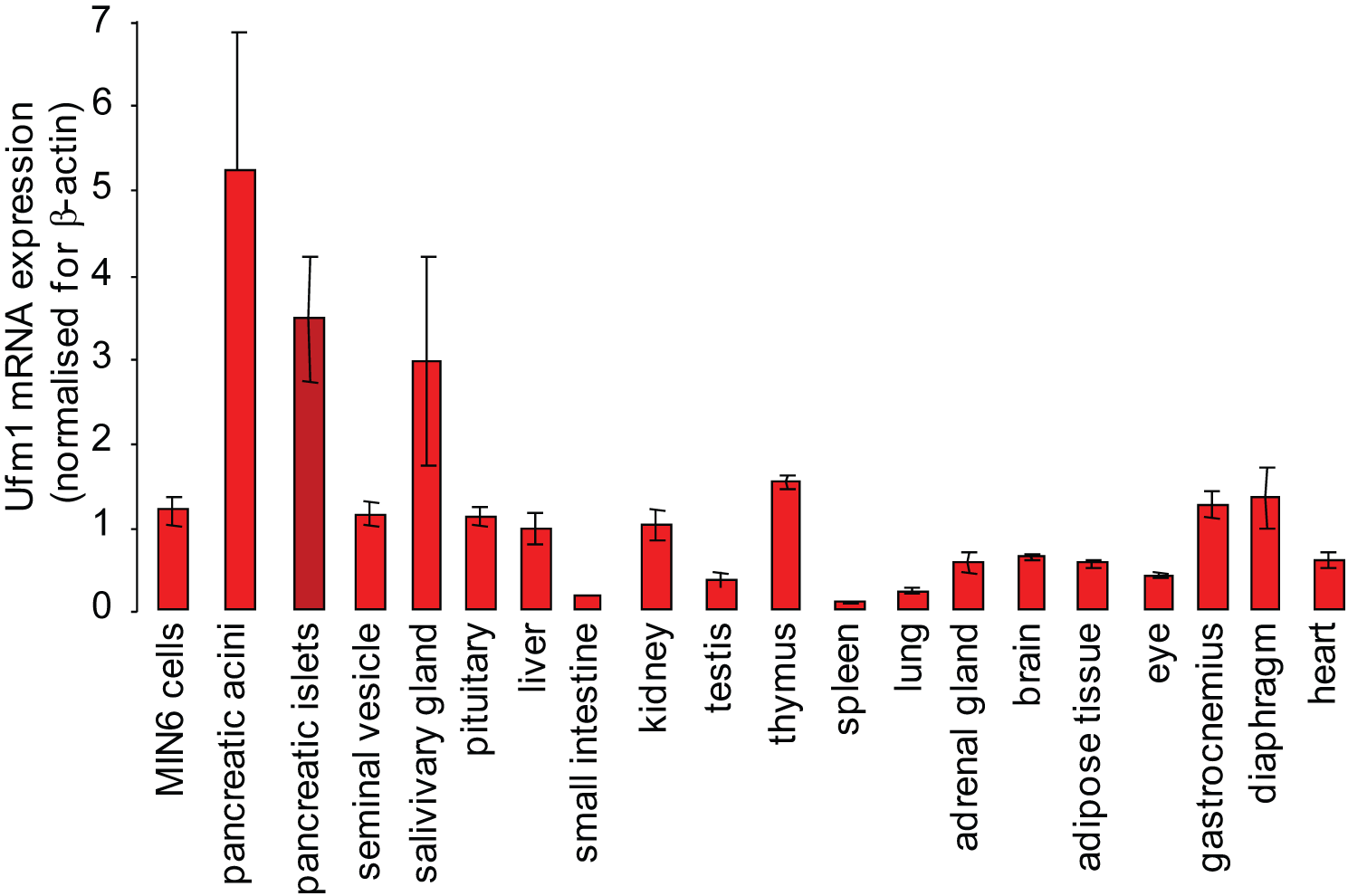

Supplement: Figure S1 — mRNA expression of Ufm1 in different mouse tissues, measured via QPCR and normalised for β-actin. Data are means±SD, n≥3. (TIF) [file pone.0018517.s001.tif]

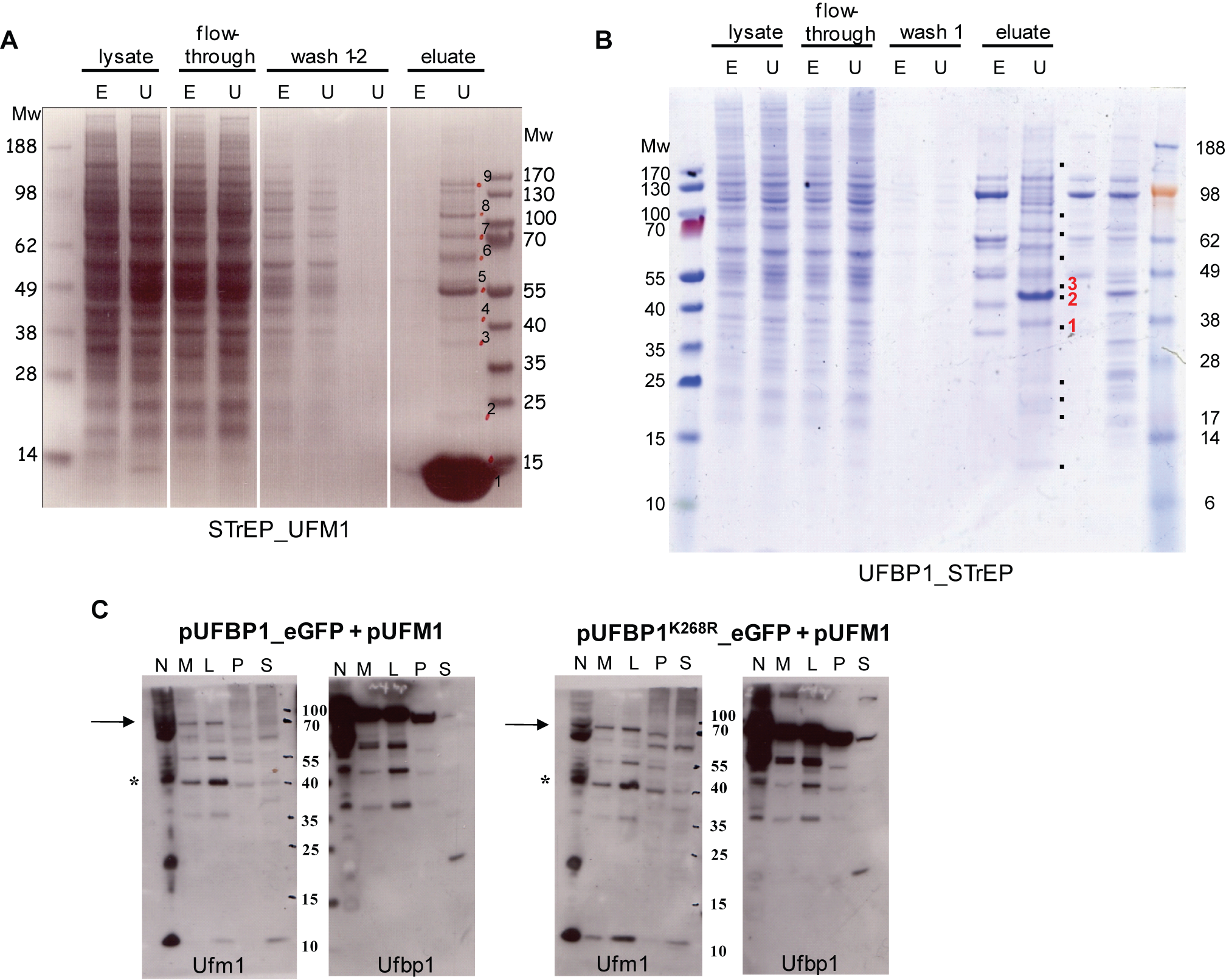

Supplement: Figure S2 — Coomassie staining of the different fractions during STrEP-tag affinity purification of UFM1 (A) and UFBP1 (B). Together with the eluate samples, a small aliquot was taken from the protein extract before and after binding to the beads and from the wash step. MIN6 cells were transfected with empty vector (E) or with a vector containing STrep-Ufm1 (U). Two different molecular weight markers were used. The protein fragments used for MS/MS identification are indicated and numbered. The identification of the proteins of UFM1 purification is shown in table 1, B Three distinct bands were shown to contain the UFBP1 protein. Fragment 1: the presence of both UFBP1 and G3P (glyceraldehyde 3-phosphate dehydrogenase, mass: 35810 Da) was demonstrated. The tryptic peptide containing the unmodified K268 (sequence: IQDLLTEGTLTGVIDGGK, mass: 2044 Da) in UFBP1 was measured with confidence (delta mass of 0.01 Da). Also the tryptic peptide following the K268 residue (sequence: FIYITPEELAAVANFIR, mass: 1967 Da) was demonstrated with confidence (delta mass: 0.04 Da), supporting the idea K268 is not modified and thereby excluded as a trypsin-cleaving site. Peptide fragmentation data was generated by MS/MS analysis and confirmed the peptide identities. Fragment 2: this band is identified as UFBP1. Again the 2044 (delta: 0.01 Da) and the 1967 (delta: 0.04 Da) masses were present. MS/MS analysis confirmed the AA sequences. The elution position in the SDS-PAGE gel corresponds to the expected molecular weight of the native protein (35956 Da). Fragment 3: Two proteins were identified: PDIA6 (protein disulfide-isomerase A6) and UFBP1. The position in the SDS-PAGE gel fits perfectly with the mass of PDIA6 (48070 Da) but is about 10 kDa too high for UFBP1. However, it is in perfect agreement with a UFM1 modification (+9.1 kDa) of UFBP1. Remarkably, both peptides (2044 and 1967), reporting the unmodified K268, are now missing, C Cellular fractionation of MIN6 cells overexpressing UFBP1_eGFP+UFM1 (le [file pone.0018517.s002.tif]

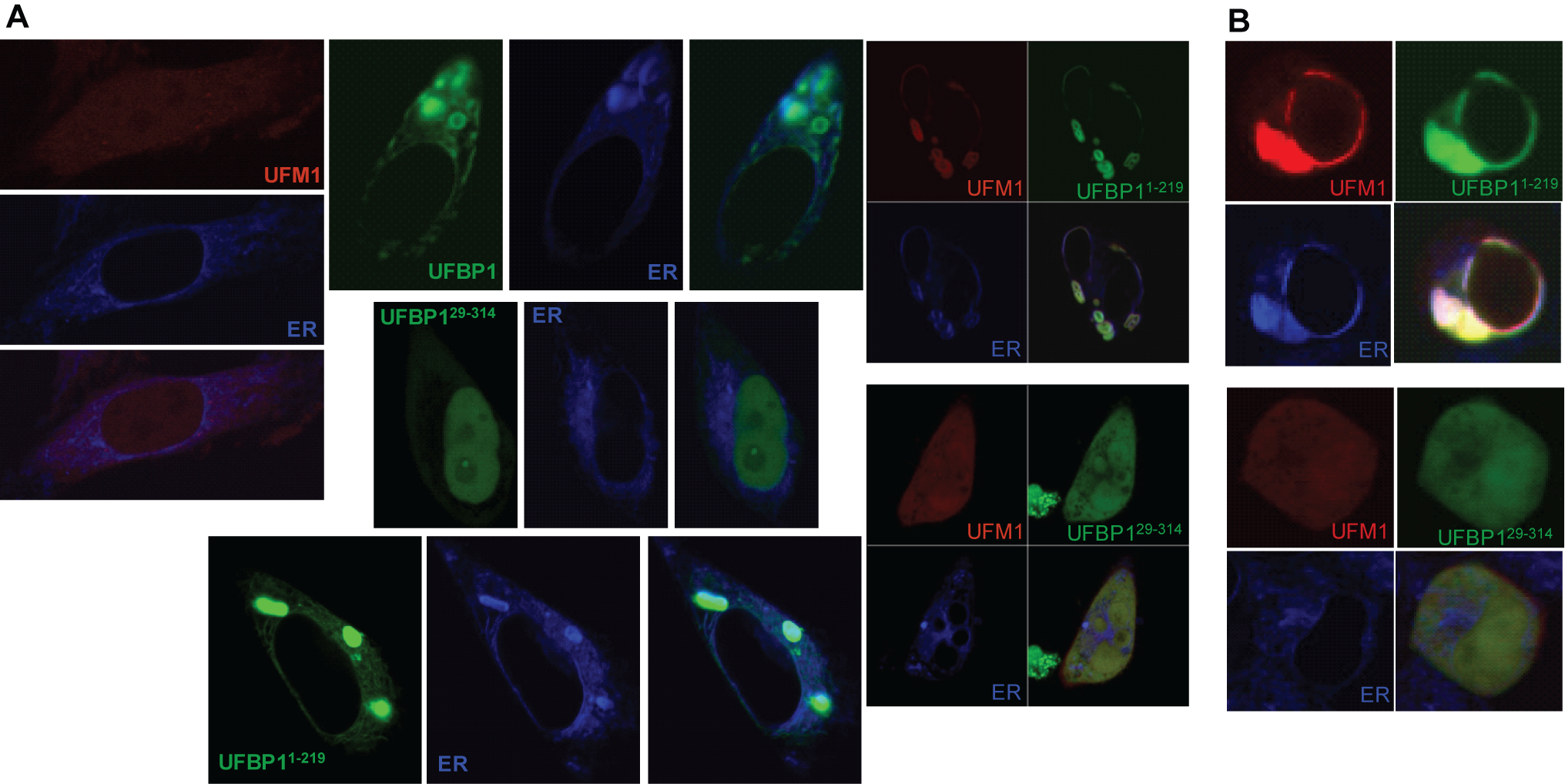

Supplement: Figure S3 — UFBP1 and UFM1 are co-localized in the ER. A HeLa cells transfected or co-transfected with different eGFP or mRFP constructs as indicated on the picture, B INS1 cells co-transfected with mRFP-UFM1 and UFBP1-eGFP as depicted. Cells were also stained with an ER-tracker (blue). Pictures were taken with a 63× objective on a Zeiss confocal microscope. (TIF) [file pone.0018517.s003.tif]

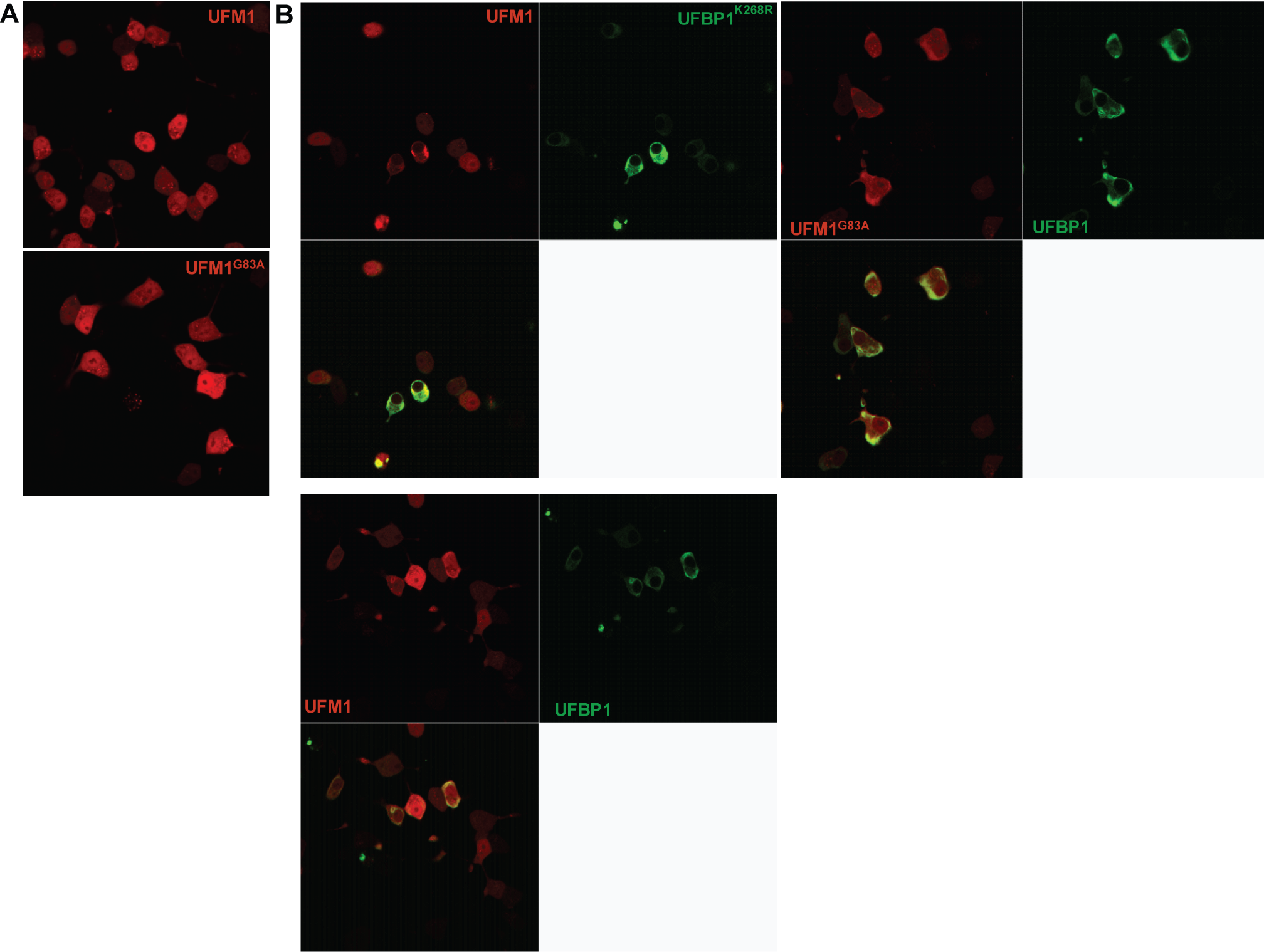

Supplement: Figure S4 — Cellular localization of UFM1 and UFBP1. A INS1-832/13 cells transfected with wild type UFM1 or UFM1G83A, they both show similar localization. B INS1-832/13 cells co-transfected with UFM1 (WT or G83A-mutant) and UFBP1 (WT or K268R-mutant) as depicted on the picture. Cells were also stained with an ER-tracker (blue). Pictures were taken with a 63× objective on a Zeiss confocal microscope. (TIF) [file pone.0018517.s004.tif]

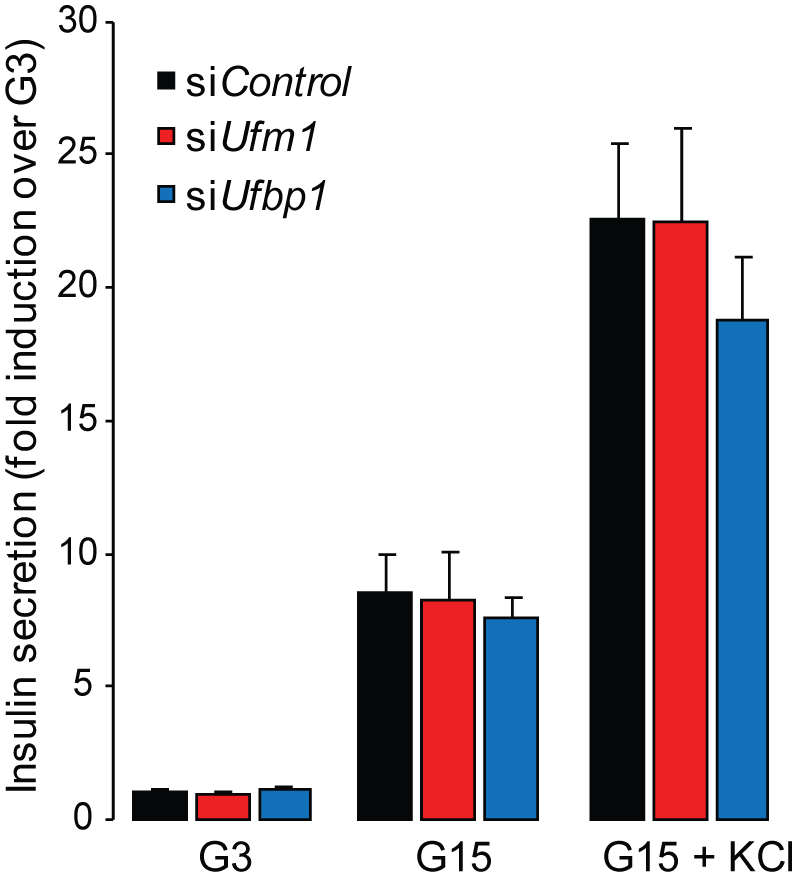

Supplement: Figure S5 — Insulin secretion is not affected by Ufm1 or Ufbp1 silencing. INS1-832/13 cells were silenced with Ufm1 (black bars) or Ufbp1 (grey bars) specific siRNA or with non-target siRNA (white bars). 48 hours after transfection, cells were incubated in medium with low (G3) or high (G15) glucose concentrations, or with high glucose concentrations together with 30 mMKCl. Data are means±SD, n = 4. (TIF) [file pone.0018517.s005.tif]

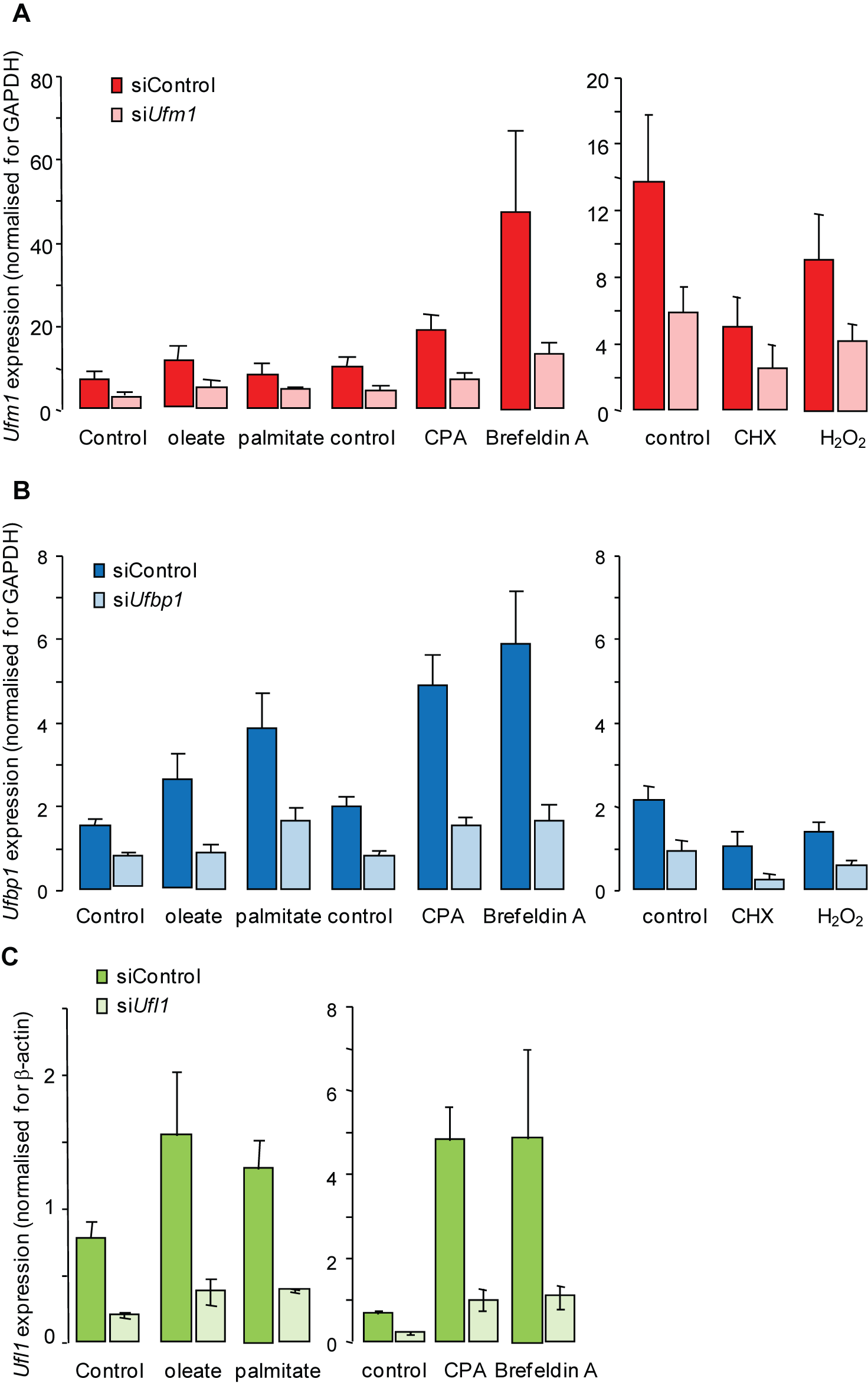

Supplement: Figure S6 — Ufm1 and Ufbp1 mRNA expression during apoptosis experiment of Figure 5 . Expression was normalized to GAPDH (Ufm1 and Ufbp1) or β-actin (Ufl1) expression. Data are means±SEM. (TIF) [file pone.0018517.s006.tif]

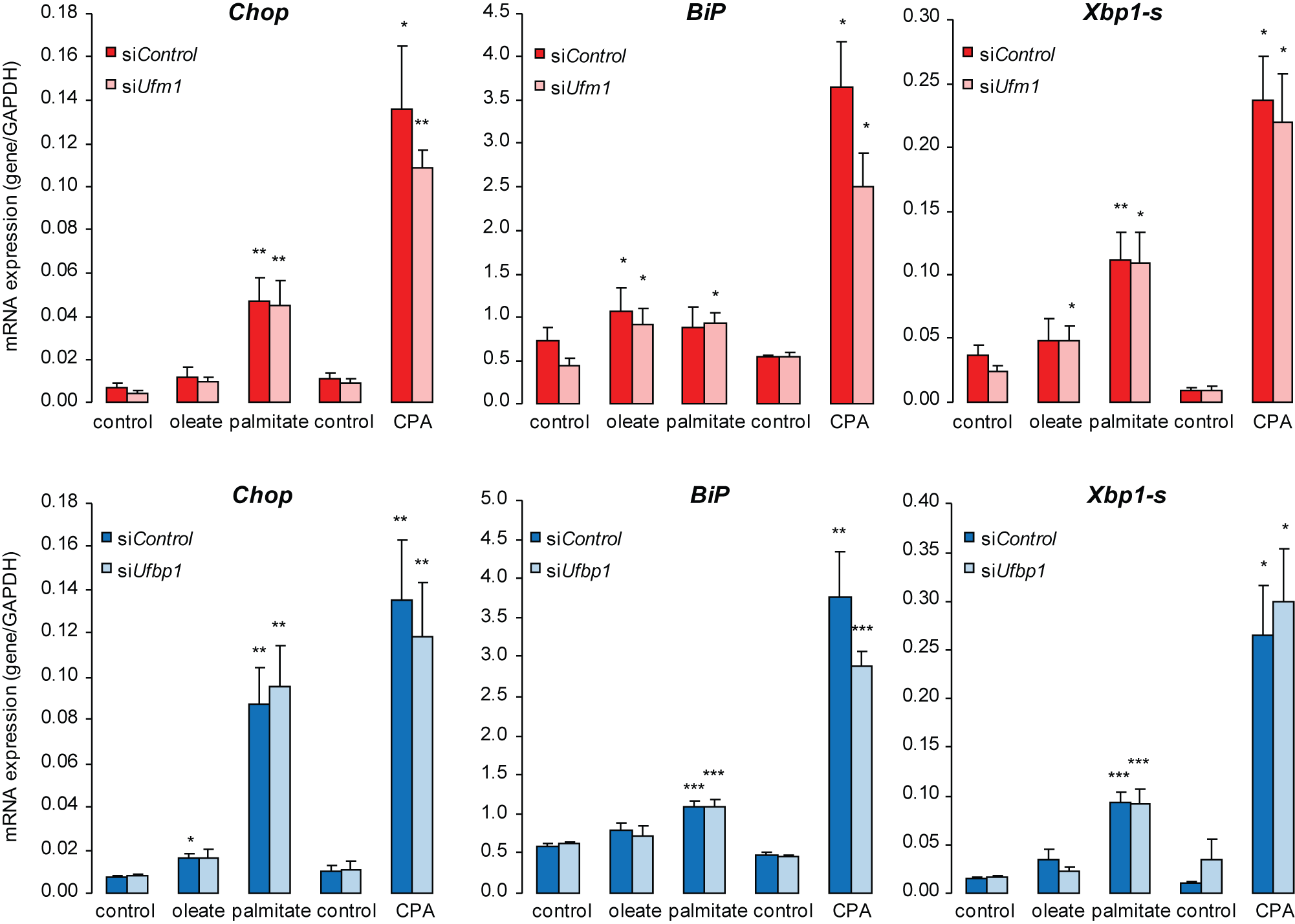

Supplement: Figure S7 — ER stress markers are not enhanced after Ufm1 or Ufbp1 silencing. INS-1E cells were transfected with siRNA against Ufm1, Ufbp1 or non-target siRNA and treated with oleate, palmitate or CPA for 14 hours. mRNA expression of BiP, Chop and Xbp1 splicing were analyzed using qPCR, and normalized for GAPDH. Data are means±SEM with n≥5, paired student t-test: *, p≤0.05; .**, p≤0.01; ***, p≤0.005. (TIF) [file pone.0018517.s007.tif]

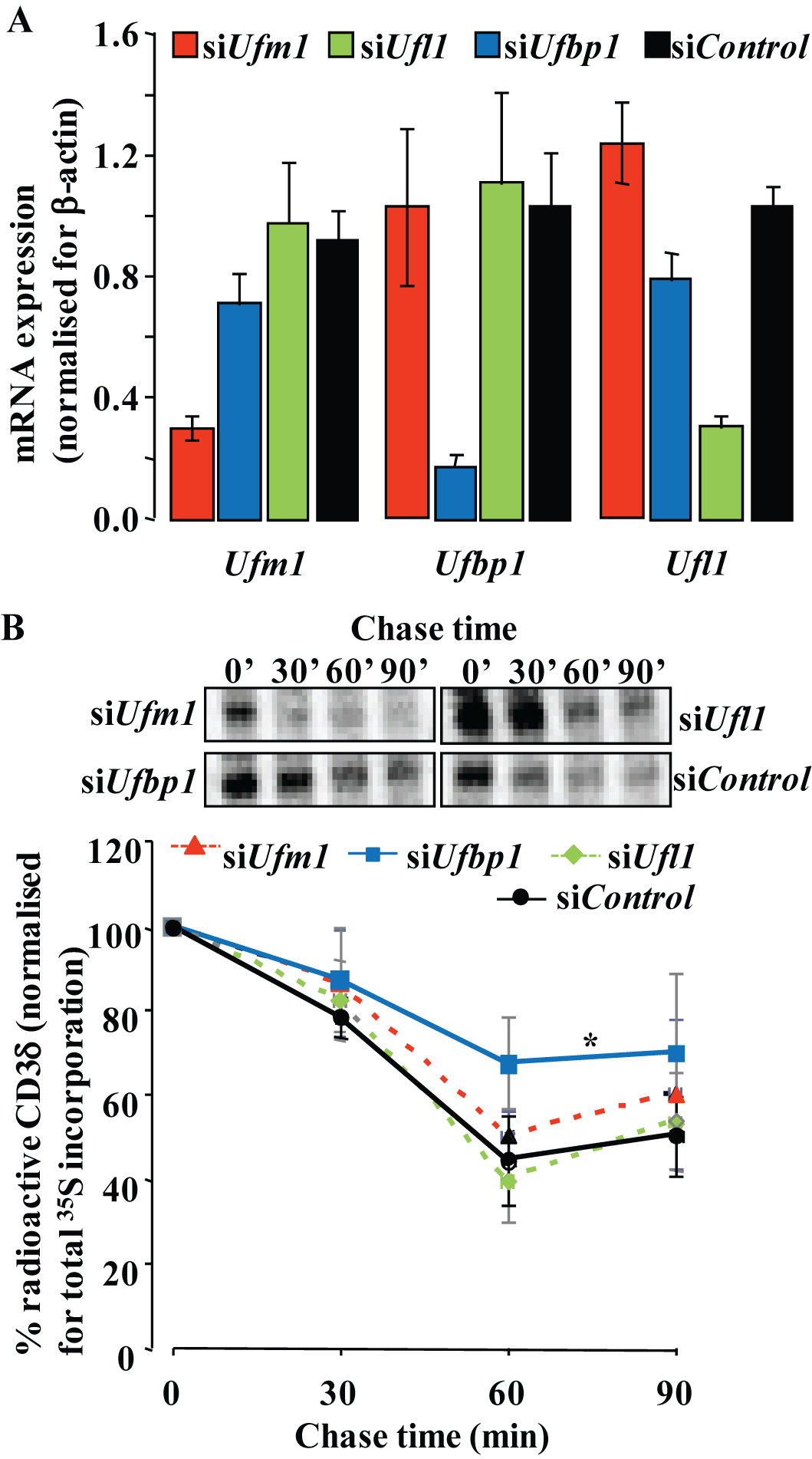

Supplement: Figure S8 — UFBP1 plays a role in ERAD. \ERAD activity was analyzed by measuring CD3δ degradation in INS1-832/12 cells transfected with siRNA against Ufm1,Ufbp1 andUfl1 and24 hours later with a CD3δ-HA expression construct, A Silencing of Ufm1 and Ufbp1 was analyzed via qPCR, B The transfected were starved for 1 hour and labeled with 35S-Met-Cys for 1 hour. After 0, 30, 60 and 90 min chase, cells were lysed and CD3δ-HA was immunoprecipitated with an HA antibody. After SDS-PAGE (upper panel), CD3δwas quantified (lower panel) and normalized for total 35S incorporation. Data are means±SEM, n = 6, *, p<0.01 with a Z-test on ratios of all time points comparing siUfbp1vssiControl. (TIF) [file pone.0018517.s008.tif]
